# The Central Circadian Clock Protein TaCCA1 Regulates Seedling Growth and Spike Development in Wheat (*Triticum aestivum* L.)

**DOI:** 10.3389/fpls.2022.946213

**Published:** 2022-07-18

**Authors:** Jie Gong, Yimiao Tang, Yongjie Liu, Renwei Sun, Yanhong Li, Jinxiu Ma, Shengquan Zhang, Fengting Zhang, Zhaobo Chen, Xiangzheng Liao, Hui Sun, Zefu Lu, Changping Zhao, Shiqing Gao

**Affiliations:** ^1^The Municipal Key Laboratory of the Molecular Genetics of Hybrid Wheat, Beijing Academy of Agriculture and Forestry Sciences, Beijing, China; ^2^Institute of Hybrid Wheat, Beijing Academy of Agriculture and Forestry Sciences, Beijing, China; ^3^National Key Facility of Crop Gene Resources and Genetic Improvement, Institute of Crop Science, Chinese Academy of Agricultural Sciences, Beijing, China

**Keywords:** CCA1, EE motif, energy metabolism, histone modification, photosynthesis, wheat

## Abstract

The biological functions of the circadian clock on growth and development have been well elucidated in model plants, while its regulatory roles in crop species, especially the roles on yield-related traits, are poorly understood. In this study, we characterized the core clock gene *CIRCADIAN CLOCK-ASSOCIATED 1 (CCA1)* homoeologs in wheat and studied their biological functions in seedling growth and spike development. *TaCCA1* homoeologs exhibit typical diurnal expression patterns, which are positively regulated by rhythmic histone modifications including histone H3 lysine 4 trimethylation (H3K4me3), histone H3 lysine 9 acetylation (H3K9Ac), and histone H3 lysine 36 trimethylation (H3K36me3). TaCCA1s are preferentially located in the nucleus and tend to form both homo- and heterodimers. *TaCCA1* overexpression (*TaCCA1-*OE) transgenic wheat plants show disrupted circadian rhythmicity coupling with reduced chlorophyll and starch content, as well as biomass at seedling stage, also decreased spike length, grain number per spike, and grain size at the ripening stage. Further studies using DNA affinity purification followed by deep sequencing [DNA affinity purification and sequencing (DAP-seq)] indicated that TaCCA1 preferentially binds to sequences similarly to “evening elements” (EE) motif in the wheat genome, particularly genes associated with photosynthesis, carbon utilization, and auxin homeostasis, and decreased transcriptional levels of these target genes are observed in *TaCCA1-*OE transgenic wheat plants. Collectively, our study provides novel insights into a circadian-mediated mechanism of gene regulation to coordinate photosynthetic and metabolic activities in wheat, which is important for optimal plant growth and crop yield formation.

## Introduction

Photosynthesis is the basis of all life on Earth; it enables plants to take the sun’s light energy and fix carbon from CO_2_ to synthesize soluble sugars and starch providing energy for their growth and development (Stitt and Zeeman, 2012; Yamori and Shikanai, 2016). Recent molecular and genetic studies have identified several factors that regulate photosynthesis processes involved in chlorophyll metabolism, photosystem assembly, and carbon fixation, all of which are crucial for plant growth and development. Pchlide oxidoreductases A and B (*PORA* and *PORB*) genes encode protochlorophyllide oxidoreductases a and b, which play roles in maintaining light-dependent chlorophyll biosynthesis in green plants (Reinbothe et al., 1996). Chlorophyll synthase (CHLG) acts in a salvage pathway for chlorophyll biosynthesis by re-esterifying the chlorophyllide a produced during chlorophyll turnover. A missense mutation in *CHLG* leads to a lower chlorophyll content and delayed chloroplast development in *Arabidopsis* and rice (Wu et al., 2007; Lin et al., 2014). Sucrose phosphate synthase (SPS) and sucrose phosphate phosphatase (SPP) are key enzymes involved in the synthesis of sucrose. Overexpressing *SPS* and *SPP* in *Arabidopsis* and hybrid poplar plants increase plant growth and biomass accumulation (Maloney et al., 2015). It has now been demonstrated that lots of genes, including *PORA*, *PORB*, *CHLG*, and *SPS*, are involved in circadian clock regulation in plants. In *Arabidopsis* and many important crops (e.g., rice and maize), biomass accumulation, photosynthesis, and seed number are increased by correct circadian regulation (Green et al., 2002; Dodd et al., 2005, 2014; Ni et al., 2009; Izawa et al., 2011; Haydon et al., 2013; Ko et al., 2016; Sanchez and Kay, 2016; Steed et al., 2021).

The circadian clock is an endogenous and autonomous timekeeping mechanism with a period of about 24 h that is found in most eukaryotic organisms. This clock allows organisms to perceive environmental cue changes (e.g., light and temperature) to help coordinate metabolic and developmental processes (Young and Kay, 2001; Harmer, 2009). In the model plant *Arabidopsis thaliana*, two partially redundant Myb-like transcription factors, LATE ELONGATED HYPOCOTYL (LHY) and CCA1, as well as the pseudo-response regulator (PRR) TIMING OF CAB EXPRESSION 1 (TOC1), represent central circadian clock components (Schaffer et al., 1998; Wang and Tobin, 1998; Strayer et al., 2000). When the endogenous clock matches the external diurnal cycle, photosynthetic activity, CO_2_ fixation rates, and fitness are increased (Dodd et al., 2005).

CIRCADIAN CLOCK-ASSOCIATED 1 is a master clock protein that mediates rhythmic expression of the clock and output genes during plant growth and development. As examples, in *Arabidopsis*, *CCA1-OE* plants abolished expression rhythms and phases of clock output genes, leading to longer hypocotyls, delayed flowering time, lower chlorophyll content, reduced CO_2_ assimilation, and reduced biomass compared to wild-type plants (Wang and Tobin, 1998; Dodd et al., 2005). In rice, OsCCA1 positively regulates expression of *TEOSINTE BRANCHED1 (OsTB1), DWARF14 (D14), and IDEAL PLANT ARCHITECTURE1 (IPA1)*, to repress tiller-bud outgrowth. Downregulating and overexpressing of *OsCCA1* increase and reduce tiller numbers, respectively. OsCCA1 also regulates *IPA1* expression to mediate panicle and grain development and could fine-tune photoperiodic flowering by directly regulating *OsGIGANTEA* (*OsGI*) (Wang et al., 2020; Sun et al., 2021). In maize and soybean, *ZmCCA1b* and four *GmLHYs* are homologous to *Arabidopsis CCA1/LHY*, playing important roles in chlorophyll metabolism, internode elongation, plant height, and drought stress responses (Ko et al., 2016; Cheng et al., 2019; Wang et al., 2021). In wheat, *TaLHY* is homologous to *Arabidopsis CCA1/LHY*, playing an important role in disease resistance against stripe rust fungus and ear heading (Zhang et al., 2015). However, the molecular basis for the clock genes to regulate wheat growth and development is largely unknown.

Wheat is one of most widely cultivated crops globally, and increasing wheat yield is known as a major pillar for producing sufficient food for a growing human population (Appels et al., 2018). So far, the molecular basis for the clock genes to regulate wheat growth and development is largely unknown. To explore the function and mechanism of action of the circadian clock in the wheat growth and development, we identified the wheat *TaCCA1* genes and characterized their roles in wheat growth and development. By generating and assessing wheat plants that overexpressing *TaCCA1*, functional assay results revealed key regulatory roles of TaCCA1 in wheat circadian rhythmicity, seedling growth, and spike development. We used DAP-seq to identify genome-wide binding sites of wheat TaCCA1 and correspondingly their direct downstream target genes. Multiple molecular biological assays showed that TaCCA1s target many output genes implicating in photosynthesis, energy metabolism, and auxin homeostasis through binding to the EE-like motif. Furthermore, TaCCA1 downregulated the expression of these output genes. To our knowledge, this investigation provides novel insights into a circadian-mediated mechanism for expressing genes in wheat to coordinate photosynthetic and metabolic activities, leading to optimal growth and development, then for the first time, establish the functions of wheat *CCA1* homoeologs and shed light to the circadian regulation in wheat.

## Materials and Methods

### Plant Materials and Growth Conditions

The wheat cultivar 73064-1, a winter wheat and widely used in the wheat research community, and the model wheat cultivar “Fielder” were used in this study. All wheat plants were grown under short- (8 h light/16 h dark, 6:00 light on) and long- (16 h light/8 h dark, 6:00 light on) day conditions with a light intensity of 500 μmol/m^2^/s and 25°C/20°C light/dark temperatures.

### Growth Trait Measurement

For aerial biomass, above 10 ground seedlings for each line were collected at 10 days after planting and then weighed after desiccation for 48 h at 80°C. Spike length (SL) and kernel number per spike (KNS) are measured as the mean of the main spikes of 10 independent plants from each line. For seed weight, 100 kernels randomly selected from each line were weighed. For kernel length (KL) and kernel width (KW), 10 kernels randomly selected from main spike of each line were measured by manual. All experiments were replicated three times, unless noted otherwise.

### Chlorophyll Content Measurement

Plant tissues’ chlorophyll content was determined using a spectrophotometer according to a previously described method, with minor modification (Wu et al., 2007). Fresh leaves of WT and *TaCCA1-7D-*OE lines were cut and immersed in 10 ml ethanol for 48 h under dark conditions, following which plant residuals were removed by centrifugation and the supernatants were analyzed with a UV-1800 Spectrophotometer (SHIMADZU, Japan) scanning at 470, 649, and 665 nm.

### Starch Content Measurement

Leaf samples were weighed and immediately frozen in liquid nitrogen. The frozen tissues were ground, mixed with a homogenization buffer [500 mM 3-(N-Morpholino) propanesulfonic Acid (MOPS) pH 7.5, 5 mM Ethylenedinitrilotetraacetic acid (EDTA), and 10% ethyl glycol] and then filtered through Miracloth (CalBiochem, San Diego, CA, United States). After centrifugation, pellets were dissolved in DMSO to extract the insoluble carbohydrate fraction. The starch content was measured from the insoluble carbohydrate fraction using a commercial assay kit according to the manufacturer’s instruction (R-Biopharm, Darmstadt, Germany).

### RNA Extraction and qPCR

For the expression analysis of the target genes, total RNA was extracted from plant tissues using an RNAprep pure Plant Kit. The expression analysis of the target genes was performed using an Eco Real-Time PCR system (Illumina, San Diego, CA, United States). Wheat *Actin* gene (*TraesCS1B02G024500*) served as an internal control for the expression studies. The primers used are shown in Supplementary Table 1.

### Sequence and Phylogenetic Analyses

The conserved domain of CCA1 proteins was predicted using the SMART software (Letunic and Bork, 2018). CCA1 protein sequences are HvCCA1 (HQ850270), BdCCA1 (LOC100838310), ZmCCA1a (ZM2G474769), ZmCCA1b (ZM2G014902), SbCCA1 (Sb07g003870), OsCCA1 (Os08g0157600), AtCCA1 (At2g46830), AtLHY (At1g01060), PnLHY1 (AB429410), PnLHY2 (AB429411), BraA.LHY.a (Bra030496), McCCA1 (AY371287), GmLCL1 (Glyma.16G017400), and GmLCL2 (Glyma.03G261800), Hv: *Hordeum vulgare*; Bd: *Brachypodium distachyon*; Zm, *Zea mays*; Sb: *Sorghum bicolor*; Os: *Oryza sativa*; At: *Arabidopsis thaliana*; Pn: *Populus nigra*; BraA: *Brassica rapa*; Mc: *Mesembryanthemum crystallinum;* and Gm: *Glycine max*. Full-length protein sequences were aligned using the Clustal W module (Larkin et al., 2007). The numbers of amino acid substitutions between each pair of LHY/CCA1 proteins were estimated by the Jones–Taylor–Thornton (JTT) model with the complete-deletion option (Jones et al., 1992). From estimated numbers of amino acid substitutions, the phylogenetic tree was reconstructed using the neighbor-joining method (Saitou and Nei, 1987). The bootstrap values were calculated with 1,000 replicates and shown next to the branches (Felsenstein, 1985). These analyses were performed using the MEGA7 software^1^ (Kumar et al., 2016).

### Chromatin Immunoprecipitation Assay Followed by qPCR

Chromatin immunoprecipitation (ChIP) assays were performed according to a previously described method (Li N. et al., 2018). Briefly, 2 g of samples were washed twice in cold phosphate-buffered saline (PBS) buffer, and proteins were cross-linked to DNA by incubating the samples with formaldehyde at a final concentration of 1% on a shaking device for 10 min at 4°C. Samples were then lysed, and chromatin was precipitated on ice. Chromatin was then sonicated to yield soluble sheared chromatin (200–500 bp). One part of the soluble chromatin was saved at −20°C for input DNA, and the remainder was used for immunoprecipitation with antibodies for H3K9Ac (CST, 9649), H3K4Me3 (CST, 9751), H3K36Me3 (CST, 4909), and normal rabbit IgG (CST, 2729). Immunoprecipitated DNA was amplified by PCR using their specific primers. PCR reactions were set up and run using the ChamQ SYBR Color qPCR Master Mix. The enrichment values were normalized to the input sample. The primers used are shown in Supplementary Table 1.

### Subcellular Localization in Wheat Protoplasts

The coding sequence (CDS) (excluding the stop codon) of *TaCCA1s* was amplified with gene-specific primers and fused to the N-terminus of *GFP* in green fluorescent protein (GFP) expression vector (*CaMV35S-GFP-NOS*). Wheat protoplasts were isolated from the mesophyll tissue of wheat seedlings and then transformed using the polyethylene glycol (PEG) transfection method (Shan et al., 2014) separately with the plasmid DNA of *35S:TaCCA1-GFP*, and *35S:GFP* control as described previously. After PEG transfection, wheat protoplasts were incubated in W5 solution in a dark chamber at 23°C for 18 h, and GFP fluorescence was monitored under a laser-scanning confocal microscope. The primers used are shown in Supplementary Table 1.

### Yeast Two-Hybrid Assays

The *TaCCA1-7A/B/D* CDSs were independently amplified and cloned into the destination vectors pGBKT7 or pGADT7. The yeast strain AH109 was transformed with these constructs using a lithium acetate transformation protocol (Yeast Protocols Handbook PT3024-1, Clontech). The positive colonies were selected SD/-Leu/-Trp (-LT) medium and then used for a growth assay on selective SD/-Leu/-Trp/-His/-Ade medium (-LTHA). The interactions were observed after 4 days of incubation at 30°C. The primers used are shown in Supplementary Table 1.

### Biolayer Interferometry Assay

The binding affinity of TaCCA1-7A/7B for TaCCA1-7D and dsDNAs with TaCCA1-7D was measured using the ForteBio Octet RED 96e instrument. The TaCCA1-7A and TaCCA1-7B were diluted into 10–630 nM. Streptavidin (SA) biosensors were prewetted and loaded with 10.0 μg/ml biotinylated recombinant TaCCA1-7D. The equilibrium, association, and dissociation circles were carried out repetitively from low to high concentration of TaCCA1-7A/7B solutions. None of the protein loaded biosensors was used as the reference sensors for background subtraction. The sensors with the fixed TaCCA1-7D protein sample were applied to measure binding affinity of biotin-labeled double-strand DNA (dsDNA). Binding affinities were calculated as the ratio of dissociation and association rate constants using the Octet Data Analysis Software 11.0. The 5′-biotin-labeled oligonucleotides used are shown in Supplementary Table 2.

### Generation of *TaCCA1*-OE Wheat Transgenic Lines

The overexpression construct was created by fusing the maize constitutive ubiquitin-1 (*ZmUBI-1*) promoter to the CDS of *TaCCA1-7D* in the binary vector *pLGY-02* (generously provided by Dr. Genying Li, Shandong Academy of Agricultural Science, Jinan, Shandong, China). The CDS of *TaCCA1-7D* was amplified with primers shown in Supplementary Table 1. The Plant Transformation Facility (Shandong Academy of Agricultural Science, Jinan, Shandong, China) transformed *pLGY-02-TaCCA1-7D* into the model wheat cultivar “Fielder” by *Agrobacterium*-mediated transformation according to their protocol.

### Recombinant Protein Expression and Purification

The CDS of *TaCCA1-7A*/*7B*/*7D* was amplified and cloned into the pMAL-c2X vector (GE Healthcare). The primers used are shown in Supplementary Table 1. After the cell culture was induced with Isopropyl-β-D-thiogalactoside (IPTG), it was incubated at 18°C overnight. Bacteria were harvested and lysed with a high-pressure cell cracker. After centrifugation, the cleared extract was diluted and loaded on amylose-coupled agarose resin columns prepared according to the manufacturer’s instruction (GE Healthcare). After columns were washed, maltose binding protein (MBP) and recombinant TaCCA1-7A/B/D were eluted and filtration by 0.22 μm filter unit (Millipore); the purified MBP and rTaCCA1-7A/B/D were aliquoted and stored at −80°C.

### DAP-Seq Assay and Data Analysis

DAP-seq assay was performed with purified MBP fusion protein. Briefly, 10 μg MBP fusion proteins were incubated with 100 μl amylose-coupled agarose resin in 1 × PBS at 4°C for 1 h. The beads were collected by centrifugation at 500 *g* for 2 min. 1 μg sonicated DNA (300–1,000 bp) were then co-incubated with the beads in 1 × PBS + 0.1% NP40 at 25°C for 30 min. DNA was then purified, and libraries were constricted with Nextera DNA Library Prep Kit (Illumina FC-121-1031). Paired raw reads were trimmed with Trimmomatic-v.0.36 with default parameters. The remaining reads were aligned to ensemble 1.0 genome using Bowtie v2.3.4 with the following parameters: “bowtie2-X1000–very-sensitive.” Aligned reads were sorted and filtered with MAPQ > 10 using SAMtools v1.9, and duplicated reads were removed using Picard version v2.16.0^2^. Model-based analysis of ChIP-seq 2 (MACS2) was used to define DNase I hypersensitive site (DHS) regions and DAP-seq peaks with the “keep-dup all” function. DAP-seq peaks were filtered with “Fold enrichment > 5 and FDR < 1e-5” and DHS peaks with “fold enrichment > 2 and FDR < 1e-2.” DAP-seq peaks, which were overlapped >1 bp with DHS (bedtools v2.29.0), were further used. Peaks located within 5 kb upstream of transcriptional start sites (TSSs) were considered promoter-TSS; peaks located within 5 Kb downstream of transcriptional terminate sites (TESs) were consider as “TES”; genes overlapped with the gene body were considered “Genic”; and the rest ones were considered “Intergenic,” and the closest genes were considered the associated genes. Gene Ontology (GO) enrichment analysis was performed using the EasyGO gene ontology enrichment analysis tool (Zhou and Su, 2007). The GO term enrichment was calculated using hypergeometric distribution with the *q*-value < 0.05. *Q*-values obtained by the Fisher’s exact test were adjusted with a false discovery rate (FDR) for multiple comparisons to detect overrepresented GO terms.

### Electrophoretic Mobility Shift Assay

Electrophoretic mobility shift assay was performed as previously described (Yang et al., 2019). The complementary oligonucleotides were annealed and labeled with DIG Gel Shift Kit (Roche). About 150 ng of MBP or TaCCA1-7D-MBP protein and 2 pM DIG-labeled probes were incubated in a 10 μl reaction mixture for 1 h on ice and then separated by 6% polyacrylamide gel in 0.5 × TBE buffer (pH 8.0) at 80 V for about 90 min. For competition assays, 10-, 20-, or 50-fold more non-labeled competitor DNA was mixed in the reaction before addition of the labeled probe. The oligonucleotides used are shown in Supplementary Table 2.

## Results

### Identification of *TaCCA1* Genes in Bread Wheat

The CCA1 is a core component of the plant circadian clock and has been reported to play essential roles in regulating plant growth and development in *Arabidopsis*. To characterize the function of CCA1 ortholog in wheat, we identified three highly conserved homoeologous genes *TaCCA1-7A* (i.e., *TraesCS7A02G299400*), *TaCCA1-7B* (*TraesCS7B02G188000*), and *TaCCA1-7D* (*TraesCS7D02G295400*) in bread wheat genome. Further gene structure analysis revealed that the CDS of *TaCCA1-7A*, *TaCCA1-7B*, and *TaCCA1-7D* were 2,154, 2,136, and 2,157 bp in length, and they all contained 6 exons and 5 introns (Supplementary Figure 1A). The predicted TaCCA1-7A, TaCCA1-7B, and TaCCA1-7D proteins had 717, 711, and 718 amino acids, respectively, and all included a conserved SANT (SWI3, ADA2, N-CoR, and TFIIIB) DNA-binding domain at the N terminus (Supplementary Figure 1B). Phylogenetic analysis of CCA1 homologs and related sequences in angiosperms suggested high conservation of protein sequences in both monocot and eudicot species (Supplementary Figure 2A). Domain-level analysis also revealed that the SANT DNA-binding domain of the TaCCA1 proteins was more similarly to monocot SANT domains compared with eudicots (Supplementary Figure 2B).

### *TaCCA1* Genes Exhibit Diurnal Expression Patterns

**FIGURE 1:**
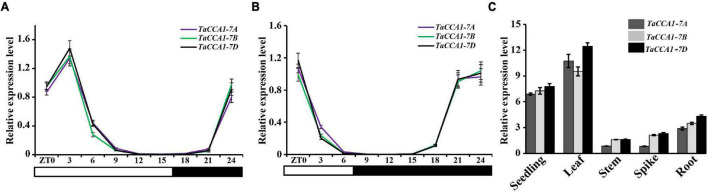
Temporal and spatial expression of *TaCCA1* homoeologs. **(A,B)** qPCR analysis of relative expression levels of *TaCCA1-7A*, *TaCCA1-7B*, and *TaCCA1-7D* every 3 h in a 24-h period (light/dark cycle is shown below the histogram) in hexaploid wheat 73064-1 under short-**(A)** and long-day **(B)** conditions. **(C)** qPCR analysis of *TaCCA1* homoeologs relative expression levels in the seedling, leaf, stem, panicle, and root. Leaf, stem, panicle, and root are from booting stage wheat plants (line 73064-1) at ZT3. Values are means ± SD (*n* = 3).

To characterize the potentially functional conservation of *TaCCA1* genes in the circadian clockwork, we measured the expression level of three *TaCCA1* genes in wheat seedlings growing under both short- and long-day conditions. Seedlings were collected every 3 h from 6:00 a.m. (at dawn, Zeitgeber time 0, ZT0) over a 24-h period, and all three *TaCCA1* genes showed typical diurnal expression patterns under both short- and long-day conditions (Figures 1A,B). The transcripts of these genes began to increase gradually at midnight (ZT15) and reached peaks of diurnal rhythms around at dawn (ZT0) under short-day condition and early morning (ZT3) under long-day condition.

We next investigated the tissue-specific expression pattern of *TaCCA1* genes in wheat at ZT3 grown under long-day condition. All three *TaCCA1* genes exhibited similar expression patterns, with a strong accumulation in green tissues (Figure 1C), which were mostly the same as those observed in *LHY/CCA1* of *Arabidopsis* and *OsCCA1* of rice (Lu et al., 2009; Sun et al., 2021). Although *TaCCA1-7A*, *TaCCA1-7B*, and *TaCCA1-7D* all expressed in wheat seedlings at ZT3, *TaCCA1-7D* showed a relatively higher expression level than the other two homoeologs. Moreover, a similar expression profile for *TaCCA1s* was evident when we examined publicly available wheat expression profiling data (Ramirez-Gonzalez et al., 2018) (Supplementary Figure 3).

Collectively, these results support that the wheat *CCA1* genes exhibit conserved diurnal expression patterns and are expressed at significantly higher levels in the green tissues than the other tissues. As green tissues function in capturing energy from the sun and converting the energy into sugars and carbohydrates for plant growth and development, we speculated a potential role of *TaCCA1s* in wheat growth and development.

### Diurnal Expression of *TaCCA1* Genes Is Associated With Histone Modification

**FIGURE 2:**
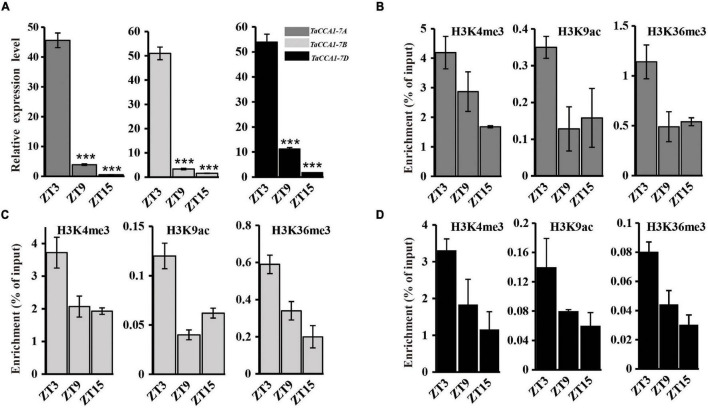
Chromatin regulation of *TaCCA1* homoeologs. **(A)** qPCR analysis the expression of *TaCCA1* homoeologs at ZT3, ZT9, and ZT15. Values are means ± SD (*n* = 3). Asterisks indicate significant differences of gene expression between ZT3 and ZT9, ZT15 using Student’s *t*-test (***P < 0.001). **(B–D)** ChIP-PCR analysis of *TaCCA1-7A*, *TaCCA1-7B*, and *TaCCA1-7D* promoter at ZT3, ZT9, and ZT15 using antibodies (Ab) against H3K4me3, H3K9Ac, and H3K36me3 in wheat line 73064-1. Values are means ± SD (*n* = 2).

Recent studies have revealed a link between circadian regulated gene expression and dynamic histone modifications. Core histones (i.e., H2A, H2B, H3, and H4) can be covalently modified at different positions with (among others) acetylation and methylation marks (Jenuwein and Allis, 2001). In *Arabidopsis*, *TOC1* expression is affected by clock-controlled cycles of histone acetylation (Perales and Mas, 2007), and both histone 3 acetylation (H3Ac) and H3K4me3 levels at the *LHY*, *CCA1*, and *TOC1* loci are positively correlated with the expression levels of their respective mRNA transcripts (Ni et al., 2009; Malapeira et al., 2012; Song and Noh, 2012; Chen and Mas, 2019). To examine the potential links between the rhythmic expression of the *TaCCA1* genes and dynamic histone modifications, we collected 14-day-old seedlings grown under long day (16 h light) conditions at ZT3, ZT9, and ZT15 and performed ChIP-qPCR and qPCR to detect the changes in histone modifications and gene expression levels, respectively. We examined chromatin changes in two different sites of the upstream regions (∼5,000 bp) of *TaCCA1* genes using antibodies against three marks for gene activation, namely, H3K4me3, H3K9ac, and H3K36me3 and then determined the expression levels of *TaCCA1* genes using qPCR. Examined together, the data revealed that the decreasing strength of *TaCCA1* genes expression observed from ZT3 to ZT9 and then to ZT15 was also reflected in decreased levels of H3K4me3, H3K9ac, and H3K36me3 marks at these three time points (Figures 2A–D and Supplementary Figure 4). These results indicate an apparent functional association between dynamic histone modifications with *TaCCA1* genes expression, indicating that *TaCCA1* gene expression is positively regulated by euchromatic histone marks H3K4me3, H3K9ac, and H3K36me3.

### The TaCCA1 Proteins Are Mainly Localized in the Nucleus and Can Form Dimers

**FIGURE 3:**
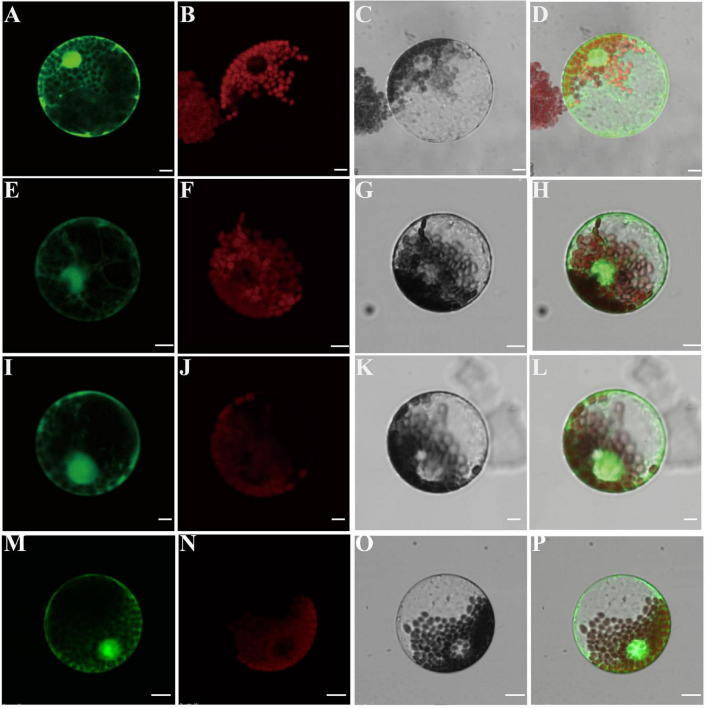
The TaCCA1 proteins are mainly localized in the nucleus. **(A–D)** Wheat protoplasts expressing green fluorescence protein (GFP) alone. **(A)** Green fluorescent protein (GFP) fluorescence. **(B)** Autofluorescence of chloroplasts. **(C)** Bright-field image of GFP. **(D)** Merged image of **(A–C)**. **(E–H)** Wheat protoplasts cell expressing the TaCCA1-7A-GFP fusion protein. **(E)** GFP fluorescence. **(F)** Autofluorescence of chloroplasts. **(G)** Bright-field image of GFP. **(H)** Merged image of **(E–G)**. **(I–L)** Wheat protoplasts cell expressing the TaCCA1-7B-GFP fusion protein. **(I)** GFP fluorescence. **(J)** Autofluorescence of chloroplasts. **(K)** Bright-field image of GFP. **(L)** Merged image of **(I–K)**. **(M–P)** Wheat protoplasts cell expressing the TaCCA1-7D–GFP fusion protein. **(M)** GFP fluorescence. **(N)** Autofluorescence of chloroplasts. **(O)** Bright-field image of GFP. **(P)** Merged image of **(M–O)**. Scale bars = 10 μm.

Recalling that the TaCCA1 proteins harbor the predicted SANT DNA binding domains, which mainly function in the nucleus, we examined their intracellular location by transiently expressing the *35S:TaCCA1s-GFP* fusion constructs in protoplasts isolated from wheat leaves. The fusion protein and a control expressing *35S:GFP* construct were monitored by confocal microscopy. Protoplasts transformed with *TaCCA1-7A-GFP* exhibited less fluorescence signals in the cytoplasm and cell membrane, majority of the fluorescence localized to the nucleus (Figures 3A–H). TaCCA1-7B and TaCCA1-7D showed the similar subcellular localization pattern with TaCCA1-7A (Figures 3I–P). These results suggest that the TaCCA1 proteins are mainly localized in the nucleus, supporting their putative function as transcription factors.

**FIGURE 4:**
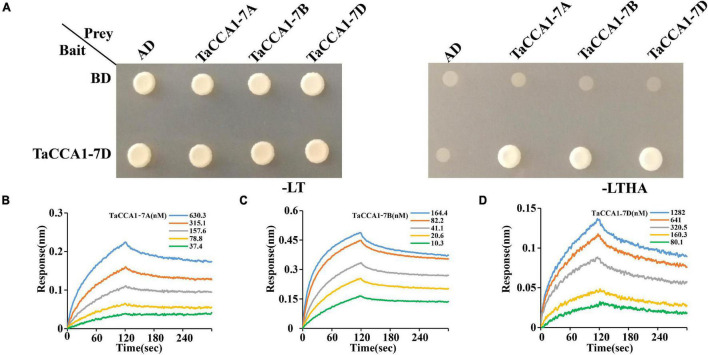
TaCCA1s can form homodimers and heterodimers *in vitro*. **(A)** Interaction analysis between TaCCA1-7D and TaCCA1-7A, as well as TaCCA1-7B and TaCCA1-7D in yeast cells. Yeast cells growing on selective media without Leu, Trp, His, and Ade (-LTHA) represent positive interactions. **(B–D)** Biolayer interferometry confirms the interaction between TaCCA1-7D with TaCCA1-7A, TaCCA1-7B, and TaCCA1-7A.

The *Arabidopsis* CCA1 can form dimers, which is functionally important for the maintenance of a roughly 24 h circadian rhythm (Lu et al., 2009; Yakir et al., 2009). We wonder whether TaCCA1 proteins take roles by forming homo- or heterodimers. Pursuing this hypothesis, we carried out yeast two-hybrid assays to characterize interactions among the TaCCA1 proteins and found that TaCCA1-7D can not only form homodimers independently but also form heterodimers with both TaCCA1-7A and TaCCA1-7B (Figure 4A). Moreover, the real-time binding graphs and kinetics parameters from the biolayer interferometry (BLI) assays further confirmed these interactions (Figures 4B–D and Supplementary Table 3). Together, these results indicate that besides the formation of homodimers as CCA1 does in *Arabidopsis*, wheat TaCCA1 proteins can also form heterodimers.

As the three *TaCCA1* homoeologs had high similarities in their expression pattern, amino acid sequences, and subcellular localization, we speculated the three *TaCCA1* homoeologs may have similar functions. Due to *TaCCA1-7D* exhibiting a relatively higher expression levels than *TaCCA1-7A* and *TaCCA1-7B* in wheat green tissues, as well as TaCCA1-7D showing closer evolutionary relationship with OsCCA1 and ZmCCA1s than TaCCA1-7A and TaCCA1-7B (Supplementary Figure 2A), it was selected for further functional analysis.

### Overexpressing *TaCCA1-7D* Represses Seedling Growth and Reduces Yield in Wheat

**FIGURE 5:**
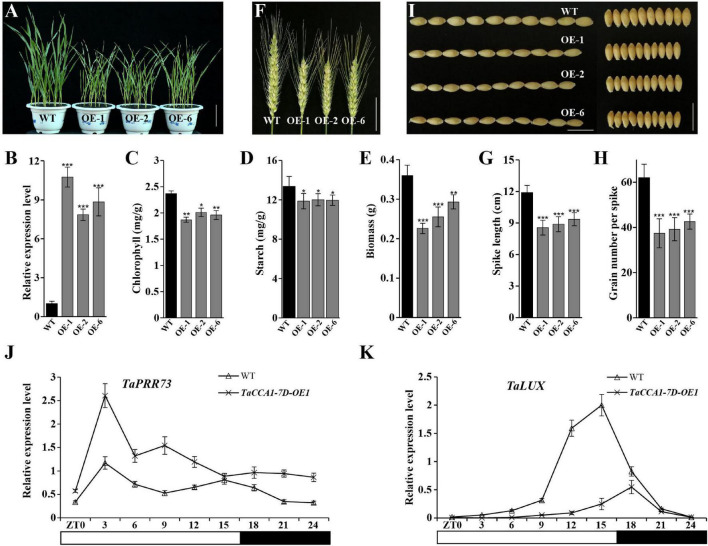
Phenotypes of *TaCCA1-7D*-OE transgenic wheat. **(A)** Representative phenotype of wild-type (WT) (Fielder) and *TaCCA1-7D-*OE plants at the seedling stage. Scale bar = 5 cm. **(B)** qPCR analysis of *TaCCA1-7D* expression in 14-day-old WT and *TaCCA1-7D-*OE plants. Values are means ± SD (*n* = 3). **(C–E)** Reduced chlorophyll accumulation, starch accumulation, and dry aerial biomass of 14-day-old WT and *TaCCA1-7D-*OE-1, OE-2, and OE-6 plants. Values are means ± SD (*n* = 10). **(F)** Representative spike phenotypes of WT and *TaCCA1-7D-*OE plants at the ripening stage. Scale bar = 5 cm. **(G)** Spike length of WT and *TaCCA1-7D-*OE plants. Values are means ± SD (*n* = 10). **(H)** Grain number per spike of WT and *TaCCA1-7D-*OE plants. Values are means ± SD (*n* = 10). **(I)** Representative grain length (left) and width (right) phenotypes of WT and *TaCCA1-7D-*OE plants at the ripening stage. Scale bar = 1 cm. **(J,K**) qPCR analysis the expression of circadian genes *TaPRR73* and *TaLUX* in WT and *TaCCA1-7D-*OE1 plants. Asterisks indicate significant differences between transgenic plants and WT at the same development stages using Student’s *t*-test (*P < 0.05, **P < 0.01, ***P < 0.001).

To explore the biological functions of *TaCCA1s* in wheat, we generated *TaCCA1-7D* overexpression (*TaCCA1-7D-*OE) plants by driving *TaCCA1-7D* under the control of a constitutive *ZmUBI-1* promoter in wheat (cv. Fielder) and obtained 14 independent transgenic lines. Three *TaCCA1-7D-*OE lines, in which the *TaCCA1-7D* expression levels were obviously higher than the WT Fielder, were selected for further analysis (Figure 5B). In greenhouse conditions, compared with WT, all the three lines of *TaCCA1-7D-*OE seedlings exhibited shorter statures and a 19–37% decrease in biomass (Figures 5A,E). Also, physiological assay results indicated that transgenic lines had lower chlorophyll and starch contents than WT, all playing important roles in growth and development in plant (Figures 5C,D). Furthermore, *TaCCA1-7D-*OE plants had reduced SLs (decreased by 20–28%) and KNS (decreased by 30–40%) compared with WT plants at the ripening stage (Figures 5F–H). In addition, *TaCCA1-7D-OE* plants also had reduced tiller number (decreased by 55%), KLs (decreased by 7–10%), KWs (decreased by 11–13%), and 100-seed weight (decreased by 19–33%) compared with WTs, which were also important yield traits (Figure 5I and Supplementary Figures 5A–D). Consistent with the higher *TaCCA1-7D* expression level, we found that the *TaCCA1-7D-OE-1* plants showed more severe phenotypes than the other two lines.

### Overexpressing *TaCCA1-7D* Disrupts Circadian Rhythmicity in Wheat

As known, CCA1 is an essential component of the circadian clock that functions to maintain circadian rhythmicity in *Arabidopsis* (Wang and Tobin, 1998). To determine the effects of *TaCCA1* overexpression on circadian rhythmicity in wheat, we measured the 24-h period time course expression of circadian clock genes including *TaPRR73* and *TaLUX ARRHYTHMO* (*TaLUX*) in WT, and *TaCCA1-7D*-OE-1 plants under long-day condition. Indeed, the rhythmic expression of these two clock genes was disrupted by constitutive expressing *TaCCA1-7D* (Figures 5J,K). Moreover, we checked the expression of *TaCCA1* homoeologs and other core clock genes in *TaCCA1-7D-*OE-1 plant at ZT3 under long-day condition. We found that the expression level of *TaCCA1*-*7A*, *TaCCA1-7B*, and endogenous *TaCCA1-7D* all reduced in these plants, indicating the potential feedback regulations of *TaCCA1* homoeologs (Supplementary Figure 6). Reduced expression of *TaTOC1* and elevated expression of *TaPRR37*, *TaPRR73*, and *TaPRR95* were also found in *TaCCA1-7D-*OE*-*1 plants (Supplementary Figure 6). Together, these results establish that *TaCCA1-7D* functions are essential in the maintenance of circadian rhythmicity in wheat.

### TaCCA1-7D Preferentially Regulates Some Downstream Denes Involved in Photosynthesis and Carbon Utilization by Binding EE-Like Motifs

**FIGURE 6:**
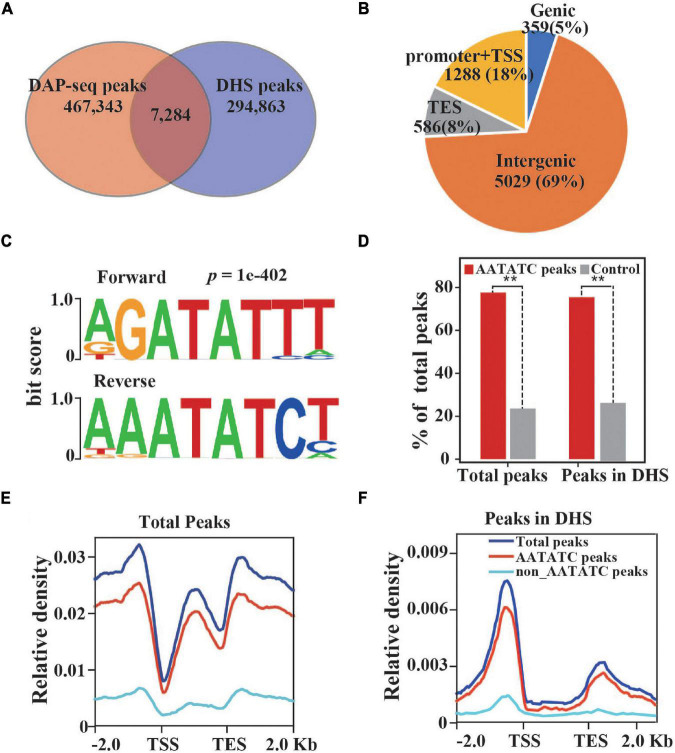
Identification of TaCCA1-7D binding loci with DNA affinity purification and sequencing (DAP-seq). **(A)** An overlap between DAP-seq peaks and DNase-I hypersensitive sites (DHSs). The overlapped peaks were used for the following analysis. **(B)** Distribution of filtered DAP-seq peaks in different genomic regions. **(C)** Enriched DNA binding elements in DAP-seq peaks. **(D)** AATATC motifs were significantly enriched in DAP-seq peaks. The shuffled genomic regions were used. **(E,F)** Distribution of DAP-seq peaks **(E)** and filtered DAP-seq peaks **(F)** in ±2 kb of genes. Asterisks indicate significant difference of enriched AATATC peaks between TaCCA1 DAP-seq and control by the Fisher’s exact test (**P < 0.01).

To explore the underlying regulation networks of TaCCA1s, the typical circadian rhythm-control transcription factor in wheat, we performed genome-wide TaCCA1-7D-binding profiles using DNA affinity purification followed by DAP-seq. Two independent DAP-seq experiments were highly correlated and showed highly Fraction of Reads in Peaks (FRiP) values (Supplementary Figure 7). The mapping statistics of DAP-seq data and distribution of DAP-seq peaks are shown in Supplementary Tables 4, 5. Transcription factor binding events usually happen in the accessible chromatin regions. Therefore, we further identified 7,284 DAP-seq peaks located in the DHSs from a previous study (Li et al., 2019) (Figure 6A). Totally, 69% TaCCA1-binding sites were located in the intergenic regions, 18% in promoter-TSS, 8% in TES, and 5% in gene bodies (Figure 6B). *De novo* motif analysis found a top-scoring motif (DRATATCH) that is similar in sequence to the well-known, clock-associated, “evening element (EE)” motif (AAATATCT) (Figure 6C). Both the total DAP-seq peaks and the DHS-filtered TaCCA1-binding sites enriched AATATC motifs (Figure 6D), suggesting the high quality of DAP-seq peaks. We further checked the profiling of TaCCA1-binding sites from 2-kb upstream of TSS to 2-kb downstream of TES, which showed TaCCA1 binding sites were mostly enriched in the 1-kb upstream of TSS and 1-kb downstream of TES (Figures 6E,F).

**FIGURE 7:**
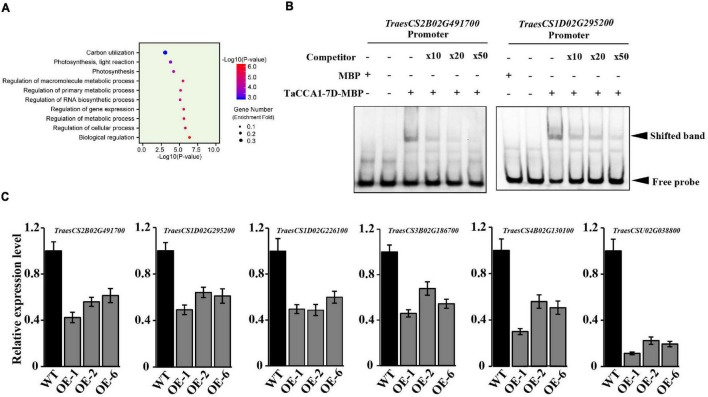
Wheat TaCCA1 regulates genes involved in photosynthesis, energy metabolism, and auxin synthesis and transport *via* binding to EE-like motifs. **(A)** Functional enrichment analysis against GO Slim of the 15,675 putative CCA1 targets identified with a peak within 5 kb of the promoter and transcriptional start site (TSS). **(B)** Electrophoretic mobility shift assay (EMSA) analysis of TaCCA1-7D binding to the EE-like motif in *TraesCS2B02G491700* and *TraesCS1D02G295200* promoter. Lane 1, labeled probe incubated with MBP; lane 2, only labeled probe loaded; lane 3, labeled probe incubated with recombinant MBP-TaCCA1-7D; lanes 4 to 6, excessive unlabeled probe was added as a competitor with the following competitor: probe ratios: 10 (lane 4), 20 (lane 5), and 50 (lane 6). **(C)** qPCR analysis of relative expression levels (means ± SEM, *n* = 3) of EE-motif-containing genes *TraesCS2B02G491700, TraesCS1D02G295200, TraesCS1D02G226100, TraesCS5D02G464800, TraesCS4B02G130100*, and *TraesCSU02G038800* in wild-type (Fielder), and *TaCCA1-7D-OE* lines grown under LD at ZT6.

Furthermore, we used the GO enrichment analysis tool catalog DAP-seq peaks to enable exploratory analysis about the potential biological impacts of TaCCA1-mediated transcriptional regulation. GO analysis indicated a strong enrichment for genes with predicted functions relating to carbon utilization, photosynthesis, and metabolic process (Figure 7A). Consistent with the relatively high levels of *TaCCA1* expression in green tissues (Figure 1C), this result emphasizes that the transcriptional regulation activity of TaCCA1-7D, and potentially the other TaCCA1 proteins, might play an important role in photosynthesis and energy metabolism.

Finally, BLI and EMSA assays were used to further confirm the binding of TaCCA1-7D at promoters of some candidate genes involved in these pathways. Specifically, we tested binding at the genes encoding CHLG (*TraesCS1D02G226100*) involved in chlorophyll synthesis, component protein of Photosystem II (*TraesCS3B02G186700*) and Photosystem II reaction center protein L (*TraesCS1D02G295200*) involved in photosynthesis, and starch synthase (*TraesCS2B02G491700*) involved in starch synthesis. EMSA assays have confirmed the binding activities of TaCCA1-7D to the promoters of *TraesCS2B02G491700* and *TraesCS1D02G295200* (Figure 7B). BLI assays also have confirmed the binding activities of TaCCA1-7D to the promoters of *TraesCS1D02G226100*, *TraesCS3B02G186700*, *TraesCS1D02G295200*, and *TraesCS2B02G491700*. TaCCA1-7D showed similar binding pattern to the promoter of these genes with single digit nano molar affinity, and the real-time binding graphs and kinetics parameters are shown in Supplementary Figure 8 and Supplementary Table 6.

To explore the underlying molecular mechanisms of *TaCCA1-7D*-*OE* phenotypes, we measured the expression of *TraesCS1D02G226100*, *TraesCS3B02G186700*, *TraesCS1D02G295200*, and *TraesCS2B02G491700* in the WT and *TaCCA1-7D-OE* lines. The results showed that the four genes all exhibited lower expression in *TaCCA1-7D-OE* lines than in WT at ZT6, consistent with the lower chlorophyll and starch contents in *TaCCA1-7D-OE* lines (Figure 7C). All these results support the notion that *TaCCA1s* take significant roles in regulating wheat seedling growth and yield-related traits through mediating transcriptional repression of a suite of genes involved in photosynthesis and energy metabolism pathways in plants. Thus, constitutive expressing *TaCCA1* in *TaCCA1-7D-OE* transgenic wheat led to repressed seedling growth and decreased yield.

## Discussion

Circadian clock regulating growth and development in *Arabidopsis* has been comprehensively surveyed, while comparatively less is known in crop species. In this study, we conducted an initial characterization of the wheat core circadian clock gene *TaCCA1s* and explored their regulation mechanisms and contributions to seedling growth and spike development in wheat, one of the most widely cultivated cereals globally. Our work establishes that the wheat TaCCA1 proteins all mainly localize to the nucleus and can dimerize into homo- and heterodimers. It has been shown that heterodimerization of central clock components contributes to the regulation of clock function in many circadian systems (Dunlap, 1999). Examples include the interaction of Period (PER) and Timeless (TIM) in *Drosophila* as a means of controlling the nuclear entry and degradation of both proteins (Edery, 1999; Meyer et al., 2006), heterodimers of the transcription factors mouse circadian locomotor output cycles kaput (mCLOCK), and brain and muscle Arnt-like protein-1 (BMAL1) in mammals accelerating their turnover, as well as E-box-dependent clock gene transcription (Reppert and Weaver, 2002; Lowrey and Takahashi, 2004) and interactions between CCA1 and LHY in *Arabidopsis* function synergistically in regulating circadian rhythms (Lu et al., 2009; Yakir et al., 2009). In this study, we demonstrated that TaCCA1 proteins all mainly localize in the nucleus and can physically interact with each other forming homo- or heterodimers, suggesting that dimerization is apparently conserved aspects in the regulation of eukaryotic circadian clocks. Nevertheless, the underlying mechanisms of dimer formation *in vivo* and functional divergence between homo- and heterodimerization of TaCCA1 proteins in wheat are still needed in future studies.

*TaCCA1-OE* transgenic wheat plants show disrupted circadian rhythmicity coupling with reduced chlorophyll and starch contents, as well as biomass at the seedling stage. Meanwhile, decreased SL and grain number per spike at the ripening stage. Consistently, in allotetraploid hybrids of the eudicot *Arabidopsis* and intraspecific hybrids of the monocot maize, altered expression of *CCA1* has been implicated in increased photosynthetic and metabolic activities. In *A. thaliana*, overexpression of *CCA1* causes disrupted circadian rhythms and reduces both photosynthesis activity and fitness (Green et al., 2002). Double-mutant *cca1 lhy* plants accumulate less starch and are unable to properly regulate the rate of starch degradation to match the length of night (Graf et al., 2010). In maize, overexpressing *ZmCCA1b* reduced chlorophyll content and plant height (Ko et al., 2016). In rice, downregulating and overexpressing *OsCCA1* increases and reduces tiller numbers, respectively. *OsCCA1* also mediates panicle and grain development and could fine-tune photoperiodic flowering. Gene expression analysis involved in photosynthesis and energy metabolism pathways in *TaCCA1-OE* transgenic implied the important regulation roles of *TaCCA1* genes. Prior to our study, it was not clear whether similar mechanism(s) may mediate the growth and development of wheat.

Mechanistically, we identified genome-wide direct targets of TaCCA1 and its binding core elements (EE-like motif) in wheat for the first time, which will be a valuable resource to systematically uncover TaCCA1s-mediated circadian outputs. We identified that TaCCA1s preferentially bound at promoters harboring the EE-like motif in wheat and confirmed the binding activities using BLI and EMSA assays. Downregulation of photosynthesis- and energy metabolism-related genes in *TaCCA1-OE* lines implies the important regulation roles of *TaCCA1* homoeologs in wheat seedling growth and spike development. Clock-related improvements in fitness results from appropriate temporal regulation of energetically costly activities, including phytohormone synthesis and signaling, growth control, metabolic activities, plant–pathogen interactions, and abiotic stress responses (Covington and Harmer, 2007; Hotta et al., 2007; Nozue et al., 2007; Covington et al., 2008; Gutierrez et al., 2008; Legnaioli et al., 2009; Wang et al., 2011; Goodspeed et al., 2012). The regulation of each of these processes is disrupted in the circadian arrhythmic *A. thaliana* lines overexpressing *CCA1*. Beyond demonstrating circadian impacts on seedling growth and spike development in wheat, our study establishes functional conservation among the *Arabidopsis*, maize, rice, and wheat CCA1 orthologs. Consistent with the regulatory role of CCA1 in *Arabidopsis* and maize, we showed that TaCCA1 can bind to the promotors of photosynthetic and metabolic activity related genes through EE-like motif. Further exploration of the functions of these EE-motif-containing CCA1 target genes will reveal the mechanisms through which circadian clocks regulate unique and complex biological pathways fine-tuning the growth and development in wheat.

In addition to the conserved mechanisms in *Arabidopsis* and rice, key genes involved in auxin homeostasis and transport were identified as a potential target of TaCCA1s. We found that the promoters of *TraesCS4B02G130100* (encoding PIN, auxin efflux carrier) and *TraesCSU02G038800* (encoding YUCCA, indole-3-pyruvate monooxygenase) harbor EE-like motifs and had apparent enrichment in our DAP-seq experiment. Furthermore, we measured the expression levels of these two genes in the WT Fielder and *TaCCA1-7D-OE* (i.e., OE-1, OE-2, and OE-6) lines. The results showed that the two genes exhibited lower expression in *TaCCA1-7D-OE* lines than in WT Fielder (Figure 7C). Phytohormones, including indole acetic acid (IAA and auxin), are key regulators in promoting spike development. IAA plays essential roles in axillary meristem (AM) initiation and outgrowth by promoting cell polarity establishment and cell elongation, resulting in a distinct plant architecture, panicle type, and grain number per spike (Yue et al., 2017; Li Y. et al., 2018). IAA is also a factor stimulating assimilate transport to developing grain (Darussalam et al., 1998). Photoassimilate and dry matter import into developing grains of wheat by affecting components of grain growth, such as cell enlargement and nutrient accumulation, are enhanced by increasing IAA levels (Hess et al., 2002). Moreover, IAA plays important roles in other plant growth stages. In *Catharanthus roseus*, IBA, IAA, and NAA significantly improved plant fresh and dry weights, total chlorophyll and carotenoids content, and net photosynthetic rate (Masidur Alam et al., 2012). In *Panax ginseng*, exogenous IAA enhanced significantly the net photosynthetic rate, stomatal conductance, and transpiration rate at all growth stages (Li and Xu, 2014). Recalling the phenotypes (especially spike-related traits) of *TaCCA1-7D-OE* lines, we thought that genes involved in wheat IAA homeostasis and transport regulated by TaCCA1 played important roles in seedling growth and spike development coupling with photosynthesis and energy metabolism pathways.

Over the past decade, the seminal findings in model plant *Arabidopsis* have been used to link circadian clock gene function to agronomic traits across many eudicots and monocots (Bendix et al., 2015). Circadian genes from crops have been reported to modulate many key agriculture traits, such as tillering and flowering in rice, heterosis in maize, nodulation in legumes, tuberization in potato, postharvest management of fruit, and vegetable storage (Yanovsky et al., 2000; Goodspeed et al., 2013; Ko et al., 2016; Kong et al., 2020; Wang et al., 2020; Sun et al., 2021). Nonetheless, there remain critical knowledge gaps related to the molecular components of circadian rhythms in important crop groups and fail to widely apply circadian studies to agricultural production (Steed et al., 2021). Based on this, what urgently needs to be solved in the future is there is a need for fundamental research in the crops to understand how the circadian clock helps crops to anticipate changes in extreme condition they encounter, as well as how innovative plant breeding may improve to produce beneficial agronomic traits.

## Conclusion

**FIGURE 8:**
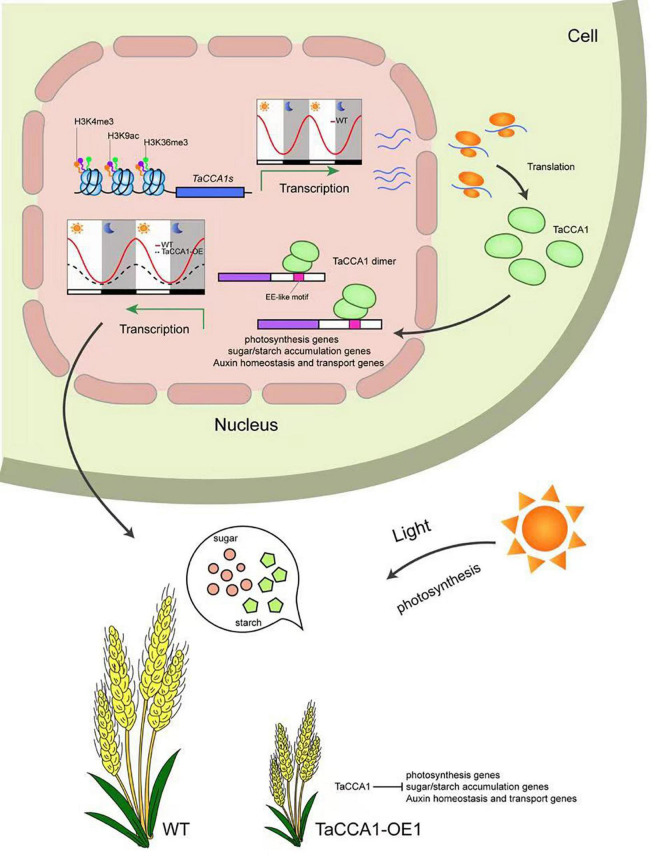
Working model of possible molecular mechanism of the central circadian clock protein TaCCA1-mediated pathways involving in seedling growth and spike-related traits in wheat. *TaCCA1* homoeologs exhibit typical diurnal expression patterns, which are positively regulated by rhythmic histone modifications. TaCCA1s are preferentially located in the nucleus and tend to form homo- or heterodimers. Overexpressing of *TaCCA1* downregulates genes implicating in photosynthesis, energy metabolism, and auxin homeostasis through binding EE-like motifs, restricting the seedling growth and development of spike-related traits.

In wheat, the central clock proteins TaCCA1s target many output genes implicating in photosynthesis, energy metabolism and auxin homeostasis through binding EE-like motif. TaCCA1s regulate the expression of genes involved in carbon assimilation and energy metabolism, which is established early in the seedling and subsequently maintained during growth. Our findings thus provide novel insights into a circadian-mediated mechanism for expressing genes in wheat to coordinate photosynthetic and metabolic activities, leading to optimal growth and development (Figure 8). In summary, our findings extend our knowledge of plant circadian clock and provide evidence for their functional roles in seedling growth and spike-related traits in the important staple crop wheat.

## Data Availability Statement

The original contributions presented in this study are publicly available. This data can be found here: NCBI, GSE200426.

## Author Contributions

JG conceived the project. JG, SG, and CZ designed the experiments. JG performed most of the experiments with help from YT, RS, YjL, YhL, JM, SZ, FZ, ZC, XL, and HS. ZL performed the DAP-seq experiment and data analysis. JG and SG analyzed the data and wrote the article. All authors approved the article for publication.

## Conflict of Interest

The authors declare that the research was conducted in the absence of any commercial or financial relationships that could be construed as a potential conflict of interest.

## Publisher’s Note

All claims expressed in this article are solely those of the authors and do not necessarily represent those of their affiliated organizations, or those of the publisher, the editors and the reviewers. Any product that may be evaluated in this article, or claim that may be made by its manufacturer, is not guaranteed or endorsed by the publisher.

## Footnotes

1
http://www.megasoftware.net/index.html

2
http://broadinstitute.github.io/picard/

## References

[B1] Appels R. Eversole K. Feuillet C. Keller B. Rogers J. Stein N. (2018). Shifting the limits in wheat research and breeding using a fully annotated reference genome. *Science* 361:eaar7191. 10.1126/science.aar7191 30115783

[B2] Bendix C. Marshall C. M. Harmon F. G. (2015). Circadian clock genes universally control key agricultural traits. *Mol. Plant* 8 1135–1152. 10.1016/j.molp.2015.03.003 25772379

[B3] Chen Z. J. Mas P. (2019). Interactive roles of chromatin regulation and circadian clock function in plants. *Genome Biol.* 20:62. 10.1186/s13059-019-1672-9 30902105 PMC6429827

[B4] Cheng Q. Dong L. Su T. Li T. Gan Z. Nan H. (2019). CRISPR/Cas9-mediated targeted mutagenesis of *GmLHY* genes alters plant height and internode length in soybean. *BMC Plant Biol.* 19:562. 10.1186/s12870-019-2145-8 31852439 PMC6921449

[B5] Covington M. F. Harmer S. L. (2007). The circadian clock regulates auxin signaling and responses in Arabidopsis. *PLoS Biol.* 5:e222. 10.1371/journal.pbio.0050222 17683202 PMC1939880

[B6] Covington M. F. Maloof J. N. Straume M. Kay S. A. Harmer S. L. (2008). Global transcriptome analysis reveals circadian regulation of key pathways in plant growth and development. *Genome Biol.* 9:R130. 10.1186/gb-2008-9-8-r130 18710561 PMC2575520

[B7] Darussalam M. Cole M. A. Patrick J. W. (1998). Auxin control of photoassimilate transport to and within developing grains of wheat.pdf. *Aust. J. Plant Physiol.* 25 69–77. 10.1071/PP97080

[B8] Dodd A. N. Kusakina J. Hall A. Gould P. D. Hanaoka M. (2014). The circadian regulation of photosynthesis. *Photosynth. Res.* 119 181–190. 10.1007/s11120-013-9811-8 23529849

[B9] Dodd A. N. Salathia N. Hall A. Kevei E. Toth R. Nagy F. (2005). Plant circadian clocks increase photosynthesis, growth, survival, and competitive advantage. *Science* 309 630–633. 10.1126/science.1115581 16040710

[B10] Dunlap J. C. (1999). Molecular bases for circadian clocks. *Cell* 96 271–290. 10.1016/S0092-8674(00)80566-8 9988221

[B11] Edery I. (1999). Role of posttranscriptional regulation in circadian clocks: lessons from *Drosophila*. *Chronobiol. Int.* 16 377–414. 10.3109/07420529908998716 10442235

[B12] Felsenstein J. (1985). Confidence-Limits on Phylogenies - an Approach Using the Bootstrap. *Evolution* 39 783–791. 10.1111/j.1558-5646.1985.tb00420.x 28561359

[B13] Goodspeed D. Chehab E. W. Min-Venditti A. Braam J. Covington M. F. (2012). Arabidopsis synchronizes jasmonate-mediated defense with insect circadian behavior. *Proc. Natl. Acad. Sci. U.S.A.* 109 4674–4677. 10.1073/pnas.1116368109 22331878 PMC3311395

[B14] Goodspeed D. Liu J. D. Chehab E. W. Sheng Z. J. Francisco M. Kliebenstein D. J. (2013). Postharvest circadian entrainment enhances crop pest resistance and phytochemical cycling. *Curr. Biol.* 23 1235–1241. 10.1016/j.cub.2013.05.034 23791724

[B15] Graf A. Schlereth A. Stitt M. Smith A. M. (2010). Circadian control of carbohydrate availability for growth in Arabidopsis plants at night. *Proc. Natl. Acad. Sci. U.S.A.* 107 9458–9463. 10.1073/pnas.0914299107 20439704 PMC2889127

[B16] Green R. M. Tingay S. Wang Z. Y. Tobin E. M. (2002). Circadian rhythms confer a higher level of fitness to Arabidopsis plants. *Plant Physiol.* 129 576–584. 10.1104/pp.004374 12068102 PMC161679

[B17] Gutierrez R. A. Stokes T. L. Thum K. Xu X. Obertello M. Katari M. S. (2008). Systems approach identifies an organic nitrogen-responsive gene network that is regulated by the master clock control gene *CCA1*. *Proc. Natl. Acad. Sci. U.S.A.* 105 4939–4944. 10.1073/pnas.0800211105 18344319 PMC2290744

[B18] Harmer S. L. (2009). The circadian system in higher plants. *Annu. Rev. Plant Biol.* 60 357–377. 10.1146/annurev.arplant.043008.092054 19575587

[B19] Haydon M. J. Hearn T. J. Bell L. J. Hannah M. A. Webb A. A. (2013). Metabolic regulation of circadian clocks. *Semin. Cell Dev. Biol.* 24 414–421. 10.1016/j.semcdb.2013.03.007 23538134

[B20] Hess J. R. Carman J. G. Banowetz G. M. (2002). Hormones in wheat kernels during embryony. *J. Plant Physiol.* 159 379–386. 10.1016/j.jplph.2004.01.003 15499903

[B21] Hotta C. T. Gardner M. J. Hubbard K. E. Baek S. J. Dalchau N. Suhita D. (2007). Modulation of environmental responses of plants by circadian clocks. *Plant Cell Environ.* 30 333–349. 10.1111/j.1365-3040.2006.01627.x 17263778

[B22] Izawa T. Mihara M. Suzuki Y. Gupta M. Itoh H. Nagano A. J. (2011). Os-GIGANTEA confers robust diurnal rhythms on the global transcriptome of rice in the field. *Plant Cell* 23 1741–1755. 10.1105/tpc.111.083238 21571948 PMC3123946

[B23] Jenuwein T. Allis C. D. (2001). Translating the histone code. *Science* 293 1074–1080. 10.1126/science.1063127 11498575

[B24] Jones D. T. Taylor W. R. Thornton J. M. (1992). The rapid generation of mutation data matrices from protein sequences. *Comput. Appl. Biosci.* 8 275–282. 10.1093/bioinformatics/8.3.275 1633570

[B25] Ko D. K. Rohozinski D. Song Q. Taylor S. H. Juenger T. E. Harmon F. G. (2016). Temporal shift of circadian-mediated gene expression and carbon fixation contributes to biomass Heterosis in Maize Hybrids. *PLoS Genet.* 12:e1006197. 10.1371/journal.pgen.1006197 27467757 PMC4965137

[B26] Kong Y. M. Han L. Liu X. Wang H. F. Wen L. Z. Yu X. L. (2020). The nodulation and nyctinastic leaf movement is orchestrated by clock gene *LHY* in *Medicago truncatula*. *J. Integr. Plant Biol.* 62 1880–1894. 10.1111/jipb.12999 33405366

[B27] Kumar S. Stecher G. Tamura K. (2016). MEGA7: molecular evolutionary genetics analysis version 7.0 for bigger datasets. *Mol. Biol. Evol.* 33 1870–1874. 10.1093/molbev/msw054 27004904 PMC8210823

[B28] Larkin M. A. Blackshields G. Brown N. P. Chenna R. Mcgettigan P. A. Mcwilliam H. (2007). Clustal W and Clustal X version 2.0. *Bioinformatics* 23 2947–2948. 10.1093/bioinformatics/btm404 17846036

[B29] Legnaioli T. Cuevas J. Mas P. (2009). TOC1 functions as a molecular switch connecting the circadian clock with plant responses to drought. *EMBO J.* 28 3745–3757. 10.1038/emboj.2009.297 19816401 PMC2790485

[B30] Letunic I. Bork P. (2018). 20 years of the SMART protein domain annotation resource. *Nucleic Acids Res.* 46 D493–D496. 10.1093/nar/gkx922 29040681 PMC5753352

[B31] Li N. Wei S. Chen J. Yang F. Kong L. Chen C. (2018). OsASR2 regulates the expression of a defence-related gene, Os2H16, by targeting the GT-1 cis-element. *Plant Biotechnol. J.* 16 771–783. 10.1111/pbi.12827 28869785 PMC5814579

[B32] Li X. Xu K. (2014). Effects of exogenous hormones on leaf photosynthesis of *Panax ginseng*. *Photosynthetica* 52 152–156. 10.1007/s11099-014-0005-1

[B33] Li Y. Fu X. Zhao M. Zhang W. Li B. An D. (2018). A genome-wide view of transcriptome dynamics during early spike development in bread wheat. *Sci. Rep.* 8:15338. 10.1038/s41598-018-33718-y 30337587 PMC6194122

[B34] Li Z. Wang M. Lin K. Xie Y. Guo J. Ye L. (2019). The bread wheat epigenomic map reveals distinct chromatin architectural and evolutionary features of functional genetic elements. *Genome Biol.* 20:139. 10.1186/s13059-019-1746-8 31307500 PMC6628505

[B35] Lin Y. P. Lee T. Y. Tanaka A. Charng Y. Y. (2014). Analysis of an Arabidopsis heat-sensitive mutant reveals that chlorophyll synthase is involved in reutilization of chlorophyllide during chlorophyll turnover. *Plant J.* 80 14–26. 10.1111/tpj.12611 25041167

[B36] Lowrey P. L. Takahashi J. S. (2004). Mammalian circadian biology: elucidating genome-wide levels of temporal organization. *Annu. Rev. Genomics Hum. Genet.* 5 407–441. 10.1146/annurev.genom.5.061903.175925 15485355 PMC3770722

[B37] Lu S. X. Knowles S. M. Andronis C. Ong M. S. Tobin E. M. (2009). CIRCADIAN CLOCK ASSOCIATED1 and LATE ELONGATED HYPOCOTYL function synergistically in the circadian clock of Arabidopsis. *Plant Physiol* 150 834–843. 10.1104/pp.108.133272 19218364 PMC2689956

[B38] Malapeira J. Khaitova L. C. Mas P. (2012). Ordered changes in histone modifications at the core of the Arabidopsis circadian clock. *Proc. Natl. Acad. Sci. U.S.A.* 109 21540–21545. 10.1073/pnas.1217022110 23236129 PMC3535592

[B39] Maloney V. J. Park J. Y. Unda F. Mansfield S. D. (2015). Sucrose phosphate synthase and sucrose phosphate phosphatase interact in planta and promote plant growth and biomass accumulation. *J. Exp. Bot.* 66 4383–4394. 10.1093/jxb/erv101 25873678 PMC4493782

[B40] Masidur Alam M. Naeem M. Idrees M. Masroor M. Khan A. Moinuddin. (2012). Augmentation of photosynthesis, crop productivity, enzyme activities and alkaloids production in Sadabahar (*Catharanthus roseus* L.) through application of diverse plant growth regulators. *J. Crop Sci. Biotechnol.* 15 117–129. 10.1007/s12892-011-0005-7

[B41] Meyer P. Saez L. Young M. W. (2006). PER-TIM interactions in living Drosophila cells: an interval timer for the circadian clock. *Science* 311 226–229. 10.1126/science.1118126 16410523

[B42] Ni Z. Kim E. D. Ha M. Lackey E. Liu J. Zhang Y. (2009). Altered circadian rhythms regulate growth vigour in hybrids and allopolyploids. *Nature* 457 327–331. 10.1038/nature07523 19029881 PMC2679702

[B43] Nozue K. Covington M. F. Duek P. D. Lorrain S. Fankhauser C. Harmer S. L. (2007). Rhythmic growth explained by coincidence between internal and external cues. *Nature* 448 358–361. 10.1038/nature05946 17589502

[B44] Perales M. Mas P. (2007). A functional link between rhythmic changes in chromatin structure and the Arabidopsis biological clock. *Plant Cell* 19 2111–2123. 10.1105/tpc.107.050807 17616736 PMC1955692

[B45] Ramirez-Gonzalez R. H. Borrill P. Lang D. Harrington S. A. Brinton J. Venturini L. (2018). The transcriptional landscape of polyploid wheat. *Science* 361:eaar6089. 10.1126/science.aar6089 30115782

[B46] Reinbothe S. Reinbothe C. Lebedev N. Apel K. (1996). PORA and PORB, two light-dependent protochlorophyllide-reducing enzymes of angiosperm chlorophyll biosynthesis. *Plant Cell* 8 763–769. 10.1105/tpc.8.5.763 12239398 PMC161135

[B47] Reppert S. M. Weaver D. R. (2002). Coordination of circadian timing in mammals. *Nature* 418 935–941. 10.1038/nature00965 12198538

[B48] Saitou N. Nei M. (1987). The neighbor-joining method - a new method for reconstructing phylogenetic trees. *Mol. Biol. Evol.* 4 406–425. 3447015 10.1093/oxfordjournals.molbev.a040454

[B49] Sanchez S. E. Kay S. A. (2016). The plant circadian clock: from a simple timekeeper to a complex developmental manager. *Cold Spring Harb. Perspect. Biol.* 8:a027748. 10.1101/cshperspect.a027748 27663772 PMC5131769

[B50] Schaffer R. Ramsay N. Samach A. Corden S. Putterill J. Carre I. A. (1998). The late elongated hypocotyl mutation of Arabidopsis disrupts circadian rhythms and the photoperiodic control of flowering. *Cell* 93 1219–1229. 10.1016/s0092-8674(00)81465-8 9657154

[B51] Shan Q. Wang Y. Li J. Gao C. (2014). Genome editing in rice and wheat using the CRISPR/Cas system. *Nat. Protoc.* 9 2395–2410. 10.1038/nprot.2014.157 25232936

[B52] Song H. R. Noh Y. S. (2012). Rhythmic oscillation of histone acetylation and methylation at the Arabidopsis central clock loci. *Mol. Cells* 34 279–287. 10.1007/s10059-012-0103-5 22878891 PMC3887839

[B53] Steed G. Ramirez D. C. Hannah M. A. Webb A. A. (2021). Chronoculture, harnessing the circadian clock to improve crop yield and sustainability. *Science* 372:eabc9141. 10.1126/science.abc9141 33926926

[B54] Stitt M. Zeeman S. C. (2012). Starch turnover: pathways, regulation and role in growth. *Curr. Opin. Plant Biol.* 15 282–292. 10.1016/j.pbi.2012.03.016 22541711

[B55] Strayer C. Oyama T. Schultz T. F. Raman R. Somers D. E. Mas P. (2000). Cloning of the Arabidopsis clock gene *TOC1*, an autoregulatory response regulator homolog. *Science* 289 768–771. 10.1126/science.289.5480.768 10926537

[B56] Sun C. Zhang K. Zhou Y. Xiang L. He C. Zhong C. (2021). Dual function of clock component OsLHY sets critical day length for photoperiodic flowering in rice. *Plant Biotechnol. J.* 19 1644–1657. 10.1111/pbi.13580 33740293 PMC8384598

[B57] Wang F. Han T. Song Q. Ye W. Song X. Chu J. (2020). The Rice Circadian Clock Regulates Tiller Growth and Panicle Development Through Strigolactone Signaling and Sugar Sensing. *Plant Cell* 32 3124–3138. 10.1105/tpc.20.00289 32796126 PMC7534462

[B58] Wang K. Bu T. Cheng Q. Dong L. Su T. Chen Z. (2021). Two homologous LHY pairs negatively control soybean drought tolerance by repressing the abscisic acid responses. *New Phytol.* 229 2660–2675. 33095906 10.1111/nph.17019

[B59] Wang W. Barnaby J. Y. Tada Y. Li H. Tor M. Caldelari D. (2011). Timing of plant immune responses by a central circadian regulator. *Nature* 470 110–114. 10.1038/nature09766 21293378 PMC6601609

[B60] Wang Z. Y. Tobin E. M. (1998). Constitutive expression of the *CIRCADIAN CLOCK ASSOCIATED 1 (CCA1)* gene disrupts circadian rhythms and suppresses its own expression. *Cell* 93 1207–1217. 10.1016/s0092-8674(00)81464-6 9657153

[B61] Wu Z. Zhang X. He B. Diao L. Sheng S. Wang J. (2007). A chlorophyll-deficient rice mutant with impaired chlorophyllide esterification in chlorophyll biosynthesis. *Plant Physiol.* 145 29–40. 10.1104/pp.107.100321 17535821 PMC1976586

[B62] Yakir E. Hilman D. Kron I. Hassidim M. Melamed-Book N. Green R. M. (2009). Posttranslational regulation of CIRCADIAN CLOCK ASSOCIATED1 in the circadian oscillator of Arabidopsis. *Plant Physiol.* 150 844–857. 10.1104/pp.109.137414 19339503 PMC2689986

[B63] Yamori W. Shikanai T. (2016). Physiological functions of cyclic electron transport around photosystem i in sustaining photosynthesis and plant growth. *Annu. Rev. Plant Biol.* 67 81–106. 26927905 10.1146/annurev-arplant-043015-112002

[B64] Yang R. Li P. Mei H. Wang D. Sun J. Yang C. (2019). Fine-tuning of MiR528 accumulation modulates flowering time in rice. *Mol. Plant* 12 1103–1113. 10.1016/j.molp.2019.04.009 31059825

[B65] Yanovsky M. J. Izaguirre M. Wagmaister J. A. Gatz C. Jackson S. D. Thomas B. (2000). Phytochrome A resets the circadian clock and delays tuber formation under long days in potato. *Plant J.* 23 223–232. 10.1046/j.1365-313x.2000.00775.x 10929116

[B66] Young M. W. Kay S. A. (2001). Time zones: a comparative genetics of circadian clocks. *Nat. Rev. Genet.* 2 702–715. 11533719 10.1038/35088576

[B67] Yue W. Fulai S. Qingrong G. Yanxia Z. Nan W. Weidong Z. (2017). Auxins regulations of branched spike development and expression of *TFL*, a LEAFY-Like Gene in Branched Spike Wheat (*Triticum aestivum*). *J. Agric. Sci.* 9 27–36.

[B68] Zhang Z. Chen J. Su Y. Liu H. Chen Y. Luo P. (2015). TaLHY, a 1R-MYB transcription factor, plays an important role in disease resistance against stripe rust fungus and ear heading in wheat. *PLoS One* 10:e0127723. 10.1371/journal.pone.0127723 26010918 PMC4444181

[B69] Zhou X. Su Z. (2007). EasyGO: Gene Ontology-based annotation and functional enrichment analysis tool for agronomical species. *BMC Genomics* 8:246. 10.1186/1471-2164-8-246 17645808 PMC1940007

