# Supplementary material for: The Central Circadian Clock Protein TaCCA1 Regulates Seedling Growth and Spike Development in Wheat (Triticum aestivum L.)
Source: Front Plant Sci. 2022 Jul 18;13:946213. doi: 10.3389/fpls.2022.946213 (PMC9340162; doi:10.3389/fpls.2022.946213)
Supplement: Supplementary file 1 [file Data_Sheet_1.docx]

**SUPPLEMENTARY MATERIAL**

**Figures**


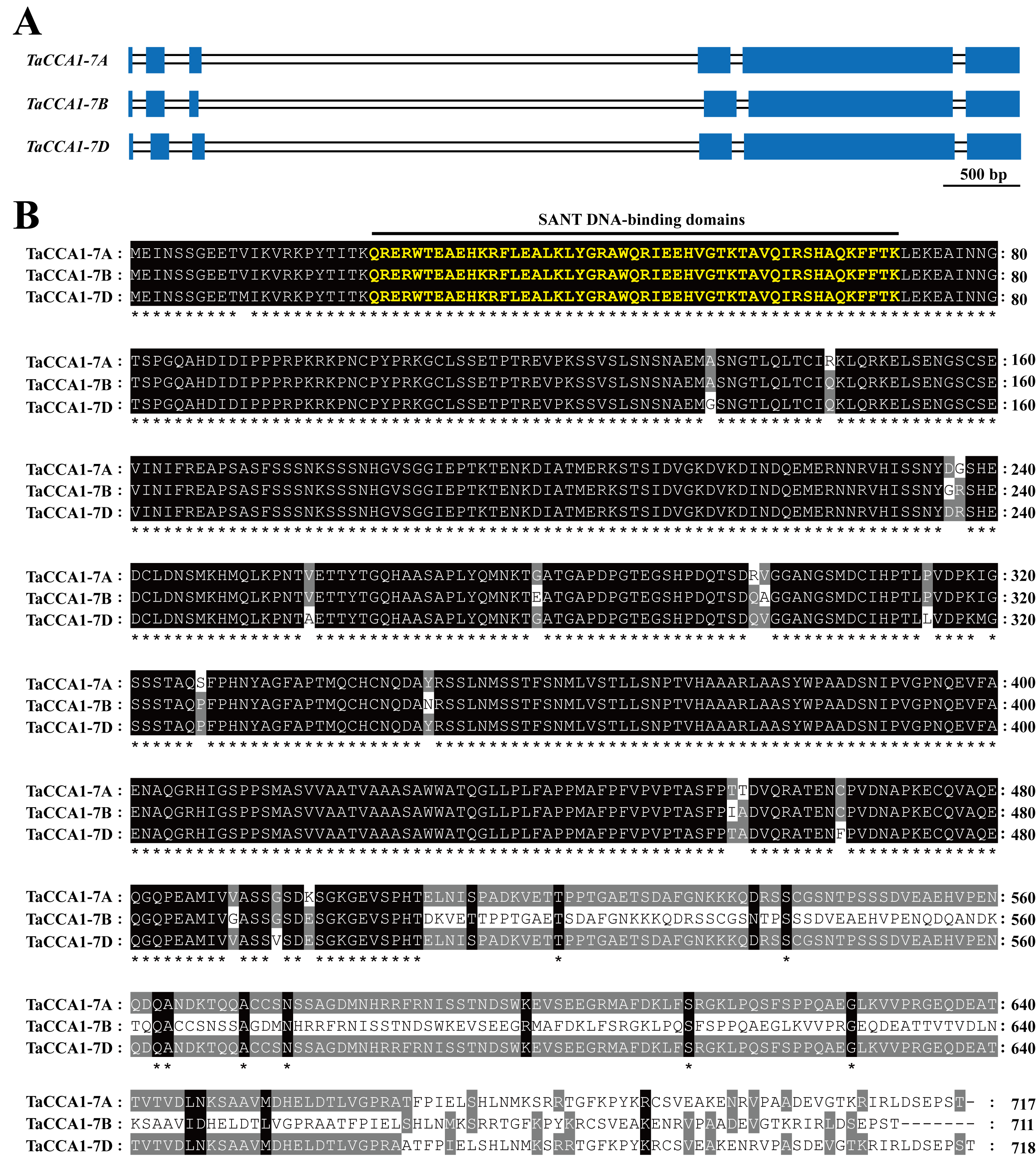


**Figure S1.** A**nalysis of *TaCCA1s* and** **TaCCA1s sequences. (A)** Schematic diagram of the three *TaCCA1* homoeologous gene structure. *TaCCA1-7A*, *TaCCA1-7B*, and *TaCCA1-7D* represent *TaCCA1* genes from the bread wheat A, B, and D genomes, Solid blocks indicate exons; lines between exons represent introns. Bar = 500 base pairs (bp). **(B)** Sequence comparison of three TaCCA1 proteins. The yellow color words represent the highly conserved SANT DNA-binding domain in three TaCCA1 homeologs.


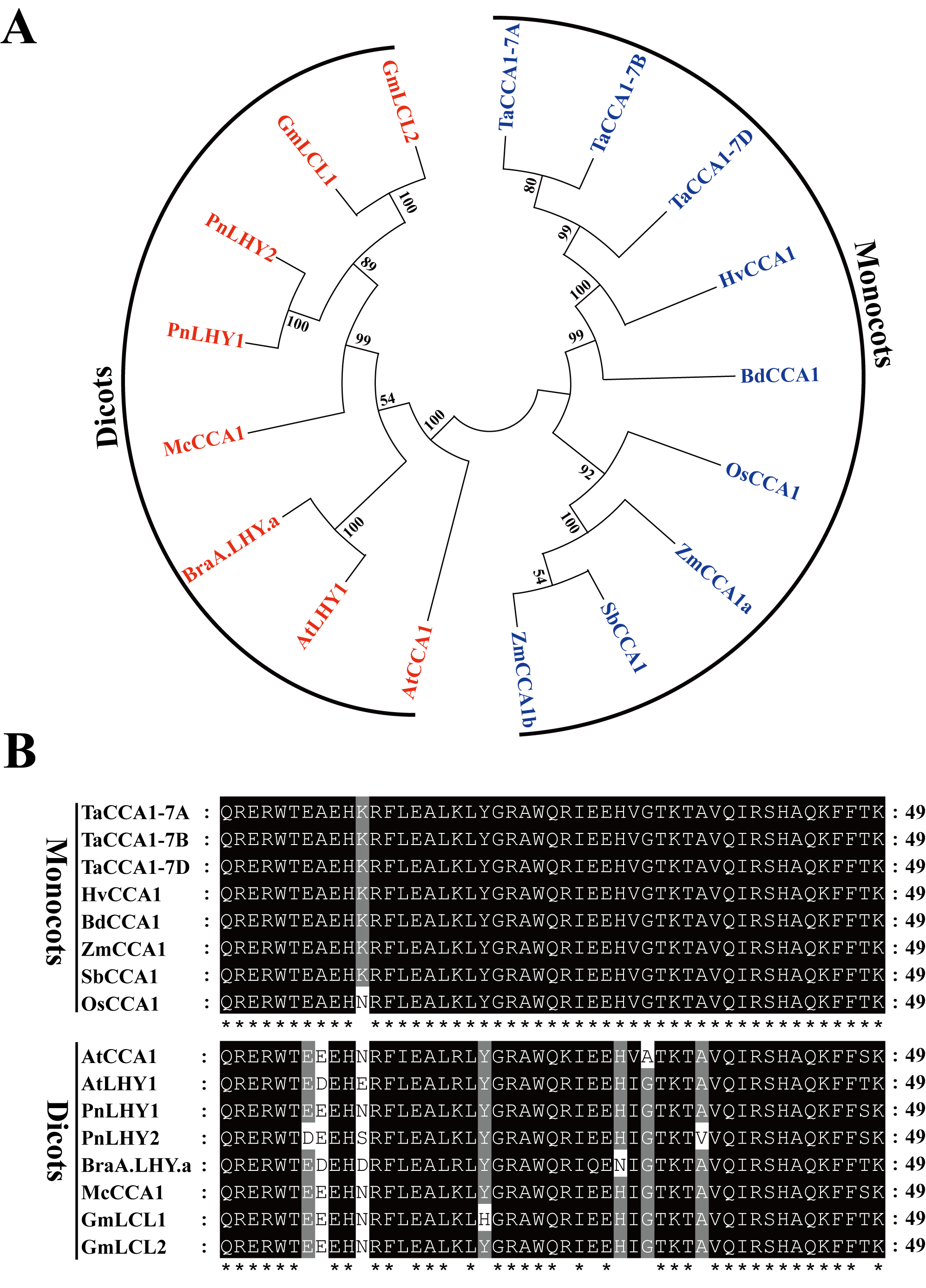


**Figure S2. Phylogenetic tree and multi-alignment of the N-terminal SANT-DNA binding domains in CCA1 homologs in plants. (A)** Phylogenetic analysis of CCA1 homologs and related sequences in angiosperms. The bootstrap values calculated with 1,000 replicates are shown next to the branches. **(B)** Multiple sequence alignment showing the N-terminal SANT-DNA binding domains of TaCCA1 and CCA1 homologs in monocots and eudicots.


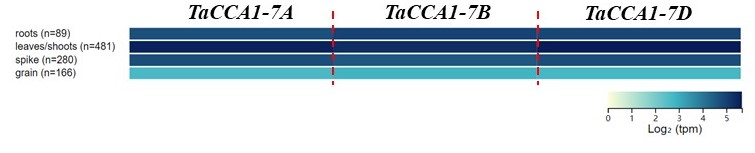


**Figure S3. Expression analysis of *TaCCA1* genes in different tissues.** Expression analysis of *TaCCA1* genes in different tissues using wheat RNA-seq data, a relatively higher expression level of *TaCCA1* genes in leaves/shoots (Ramirez-Gonzalez *et al*., 2018). Transcripts Per Million (TPM) values of target genes were logarithmically transformed for presentation and show the expression level and the dark blue modules indicate higher expression level.


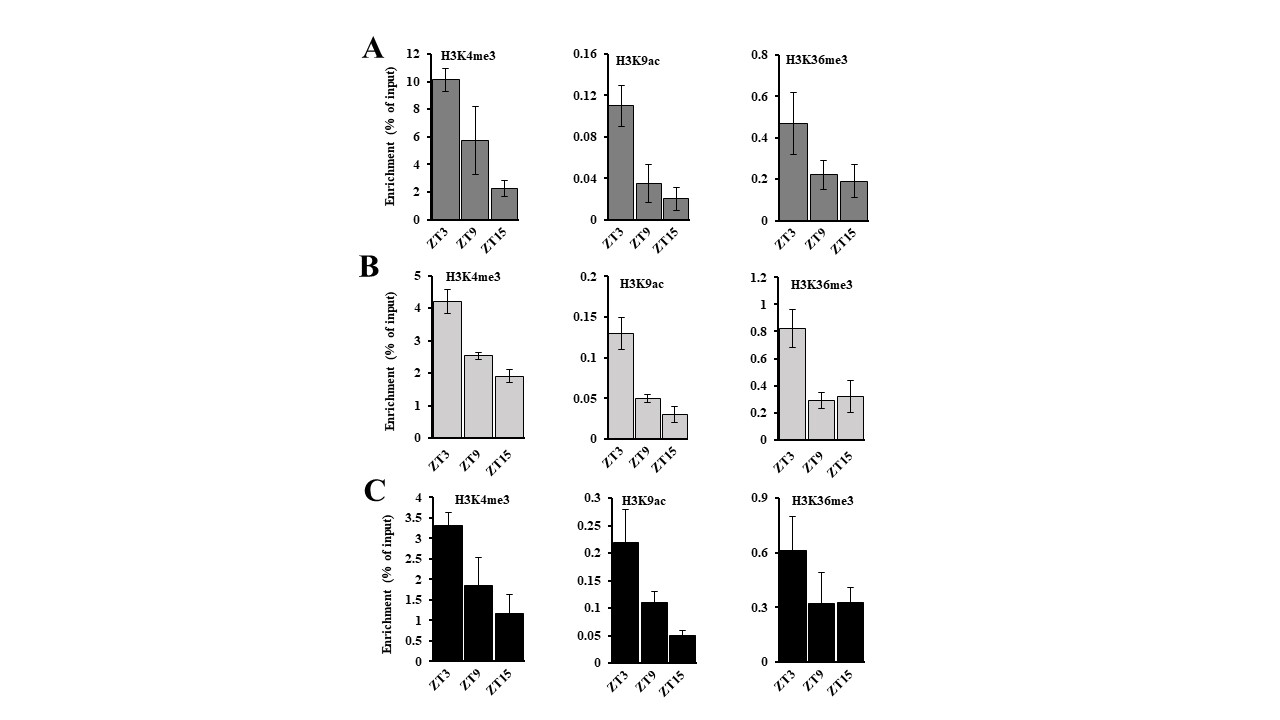


**Figure S4. Chromatin regulation of *TaCCA1* homoeologs*.*** A-C, ChIP-PCR analysis of *TaCCA1-7A* (A), *TaCCA1-7B* (B) and *TaCCA1-7D* (C) promoter at ZT3, ZT9, and ZT15 using antibodies (Ab) against H3K4me3, H3K9Ac, and H3K36me3 in wheat line 73064-1. Values are means ± SD (n = 2). The ananlysis sites in *TaCCA1-7A*, *TaCCA1-7B* and *TaCCA1-7D* promoter is different from the sites checked in Figure 3.


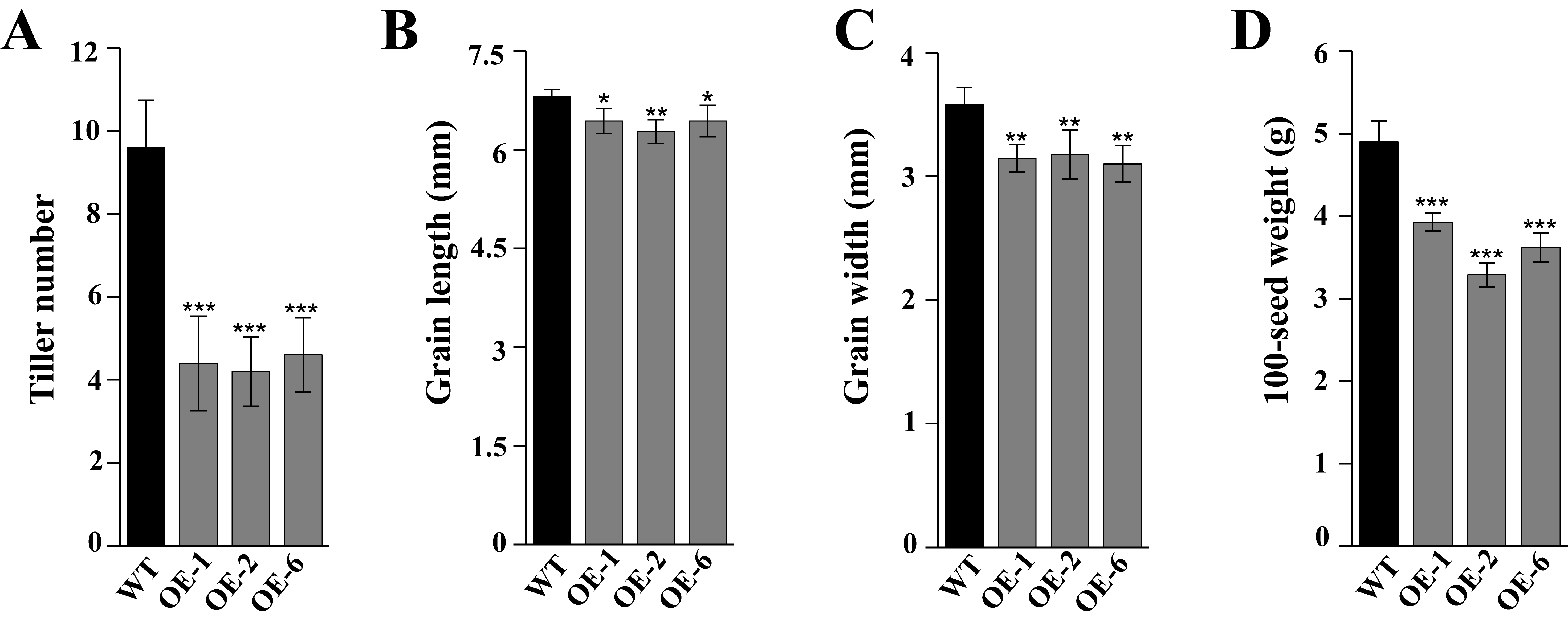


**Figure S5. Tiller number and Grain phenotypes of Wheat transgenic plants overexpressing *TaCCA1-7D*.** **(A-D)** Tiller number, Grain length, grain width, and 100-seed weight of WT and *TaCCA1-7D-*OE plants. Values are means ± SD (n = 10). Asterisks indicate significant differences between transgenic plants and WT at the same development stages using Student’s t test (**P*<0.05; ***P*<0.01; ****P*<0.001).


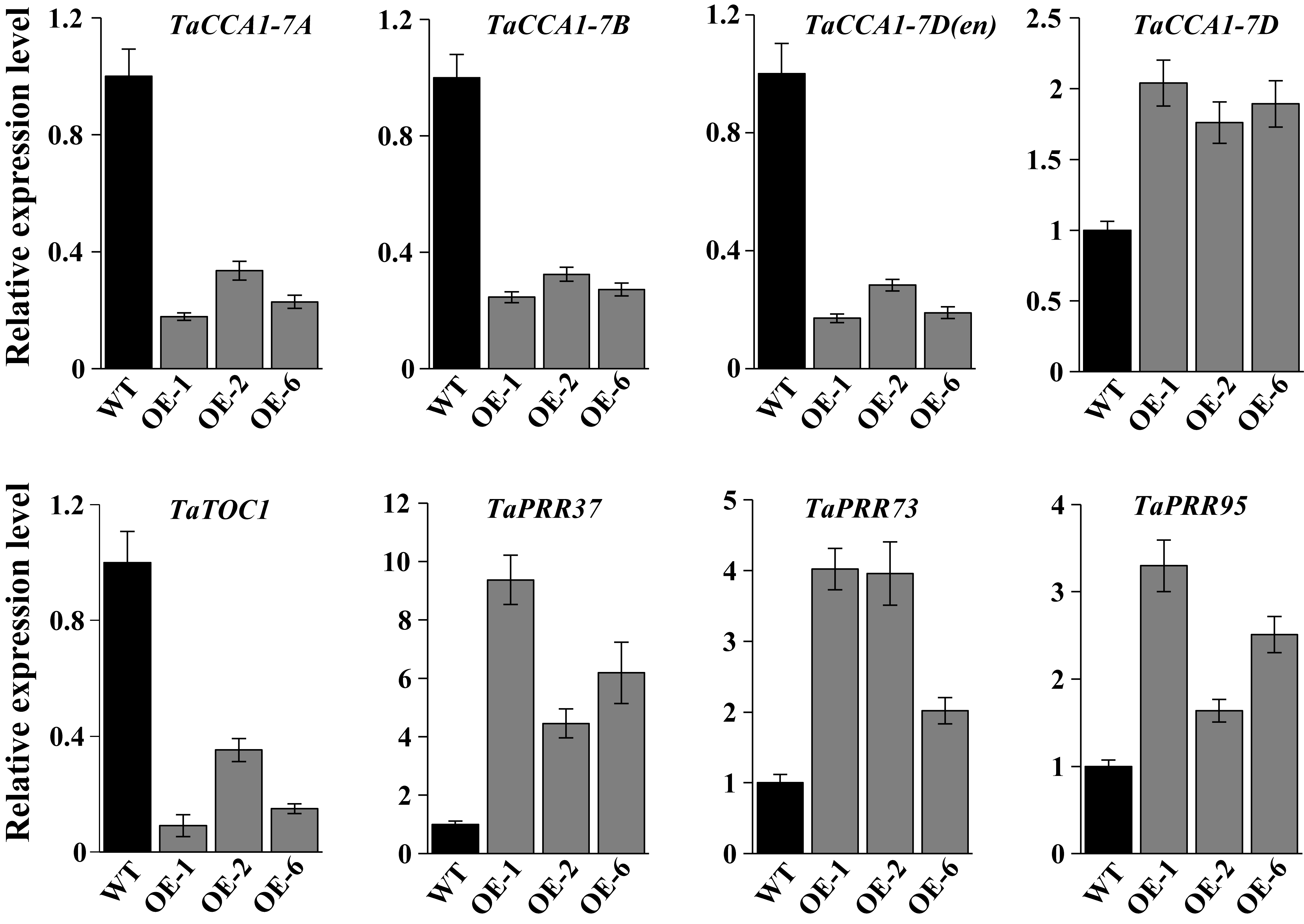


**Figure S6. Expression analysis of** **circadian clock genes in** ***TaCCA1-7D-*OE lines.** qPCR analysis of circadian clock genes expression in wild-type (Fielder) and *TaCCA1-7D-*OE-1/2/6 plants. Plants were grown under LD conditions and samples were collected at ZT3.Values are means ± SD (n = 3).


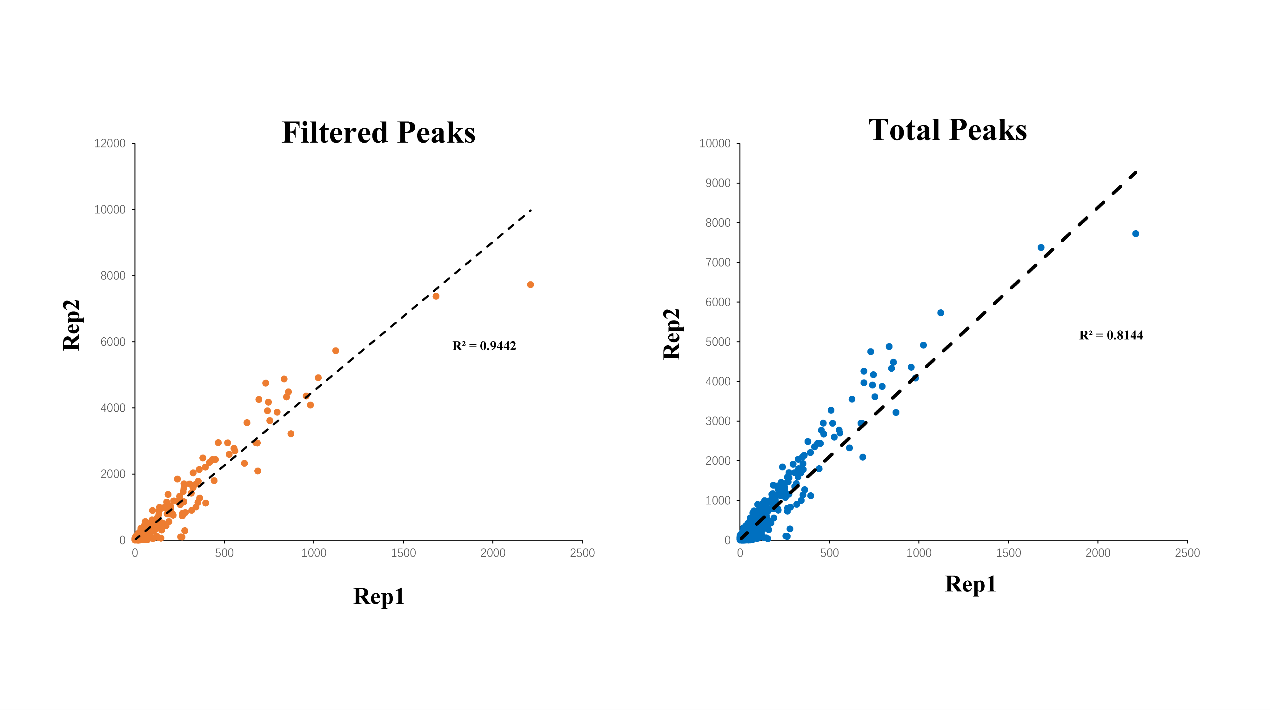


**Figure S7. Correlation of peaks between two CCA1 DAP-Seq experiments.** R^2^ values of correlation between the two DAP-seq replicates: 0.9442 of filtered peaks and 0.8144 of total peaks.


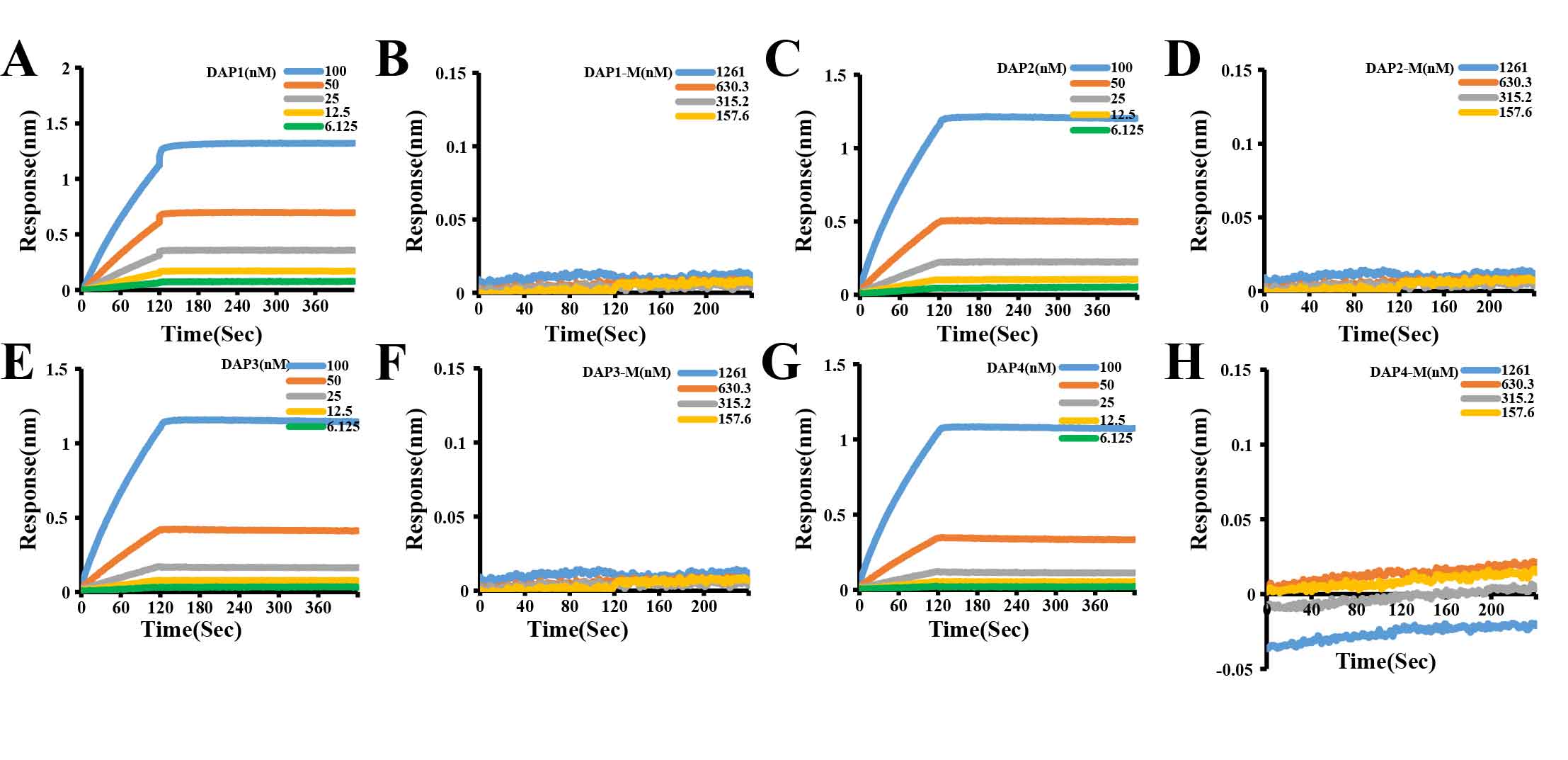


**Figure S8. Verification of TaCCA1-7D binding to promoters of Chloroplast metabolism and starch metabolism genes *in vitro* using BLI. (A-B)** BLI analysis of MBP-TaCCA1-7D fusion protein binding to DAP-1 (oligonucleotides including EE motif) and DAP-M-1 (oligonucleotides including mutated EE motif) in *TraesCS1D02G226100* promoter. Colored curves show the raw data with the concentration of DAP-1 and DAP-M-1shown at the side of the graph. **(C-D)** BLI analysis of MBP-TaCCA1-7D fusion protein binding to DAP-2 (oligonucleotides including EE motif) and DAP-M-2 (oligonucleotides including mutated EE motif) in *TraesCS1D02G295200* promoter. Colored curves show the raw data with the concentration of DAP-2 and DAP-M-2 shown at the side of the graph. **(E-F)** BLI analysis of MBP-TaCCA1-7D fusion protein binding to DAP-3 (oligonucleotides including EE motif) and DAP-M-3 (oligonucleotides including mutated EE motif) in *TraesCS2B02G419700* promoter. Colored curves show the raw data with the concentration of DAP-3 and DAP-M-3 shown at the side of the graph. **(G-H)** BLI analysis of MBP-TaCCA1-7D fusion protein binding to DAP-4 (oligonucleotides including EE motif) and DAP-M-4 (oligonucleotides including mutated EE motif) in *TraesCS3B02G186700* promoter. Colored curves show the raw data with the concentration of DAP-4 and DAP-M-4 shown at the side of the graph.

**Tables**

**Table S1. Primers used in this study.**

| **Primer Name** | | **Primer Sequence (5′to 3′)** |
| --- | --- | --- |
| **Gene cloning and vector construction** | | |
| gTaCCA1-7A-F | ATGGAGATAAATTCTTCGGGTGAGGAAACGGTG | |
| gTaCCA1-7A-R | TCACGTGGAGGGTTCGCTGTCAAGGCGAATCCT | |
| gTaCCA1-7B-F | ATGGAGATAAATTCTTCGGGTGAGGAAACGGTC | |
| gTaCCA1-7B-R | TCACGTGGAGGGTTCGCTGTCAAGG | |
| gTaCCA1-7D-F | ATGGAGATAAATTCTTCGGGTGAGGAAACAATG | |
| gTaCCA1-7D-R | TCACGTGGAGGGTTCGCTGTCAAGA | |
| TaCCA1-7D-OE-KpnI-F | gcggtaccATGGAGATAAATTCTTCGGGTGAG | |
| TaCCA1-7D-OE-SpeI-R | ttactagtcGTGGAGGGTTCGCTGTCAAGAC | |
| 16318GFP-TaCCA1-7A-HindIII-F | ggcaagcttATGGAGATAAATTCTTCGGGTGAGGAAACGGTG | |
| 16318GFP-TaCCA1-7A-BamHI-R | aatggatccCGTGGAGGGTTCGCTGTCAAGGCGAATCCTC | |
| 16318GFP-TaCCA1-7B-HindIII-F | ggcaagcttATGGAGATAAATTCTTCGGGTGAGGAAACGGTC | |
| 16318GFP-TaCCA1-7B-BamHI-R | aatggatccCGTGGAGGGTTCGCTGTCAAGGCGAATCCTC | |
| 16318GFP-TaCCA1-7D-HindIII-F | ggcaagcttATGGAGATAAATTCTTCGGGTGAGGAAACAATG | |
| 16318GFP-TaCCA1-7D-BamHI-R | aatggatccCGTGGAGGGTTCGCTGTCAAGACGAATCCTC | |
| TaCCA1-7D-BD-NdeI-F | ggccgtgcatatgGAGATAAATTCTTCGGGTGAGGAAACAATG | |
| TaCCA1-7D-BD-EcoRI-R | tatgaattcTCACGTGGAGGGTTCGCTGTCAAGACGAATCCTC | |
| TaCCA1-7A-AD-NdeI-F | ggccgtgcatatgGAGATAAATTCTTCGGGTGAGGAAACGGTG | |
| TaCCA1-7A-AD-XhoI-R | gatctcgagTCACGTGGAGGGTTCGCTGTCAAGGCGAATCCTC | |
| TaCCA1-7B-AD-NdeI-F | ggccgtgcatatgGAGATAAATTCTTCGGGTGAGGAAACGGTC | |
| TaCCA1-7B-AD-XhoI-R | gatctcgagTTACGTGGAGGGTTCGCTGTCAAGGCGAATCCTC | |
| TaCCA1-7D-AD-NdeI-F | ggccgtgcatatgGAGATAAATTCTTCGGGTGAGGAAACAATG | |
| TaCCA1-7D-AD-XhoI-R | gatctcgagTTACGTGGAGGGTTCGCTGTCAAGACGAATCCTC | |
| TaCCA1-7A-pMAL-c2X-F | AAGGATTTCAGAATTCATGGAGATAAATTCTTCGGGTGAGGAAACGGTG | |
| TaCCA1-7A-pMAL-c2X-R | CGACTCTAGAGGATCCTCACGTGGAGGGTTCGCTGTCAAGG | |
| TaCCA1-7B-pMAL-c2X-F | AAGGATTTCAGAATTCATGGAGATAAATTCTTCGGGTGAGGAAACGGTC | |
| TaCCA1-7B-pMAL-c2X-R | CGACTCTAGAGGATCCTCACGTGGAGGGTTCGCTGTCAAGG | |
| TaCCA1-7D-pMAL-c2X-F | AAGGATTTCAGAATTCATGGAGATAAATTCTTCGGGTGAGGAAACAAT | |
| TaCCA1-7D-pMAL-c2X-R | CGACTCTAGAGGATCCTCACGTGGAGGGTTCGCTGTCAAGA | |
| Restriction enzyme cutting sequences and flanking bases are lower cases | | |
| **qPCR** | | |
| TaCCA1-7A-F | CGATAAACTGTTCAGTAGAGG | |
| TaCCA1-7A-R | ATTCAATGGGAAAGGTAGCT | |
| TaCCA1-7B-F | TTCTTCGGGTGAGGAAACGGTC | |
| TaCCA1-7B-R | CACGGCCGTCTTTGTCCCAACG | |
| TaCCA1-7D-F | TTCTTCGGGTGAGGAAACAATG | |
| TaCCA1-7D-R | CACGGCCGTCTTTGTCCCAACG | |
| TaCCA1-7D-F(endogenous) | AGGAGAATAGGGTGCCGGCTT | |
| TaCCA1-7D-R(endogenous) | AACACTGCATTGCAAGGACC | |
| TaCCA1-F | ATGGAGATAAATTCTTCGGGTG | |
| TaCCA1-R | TCTTCTATGCGCTGCCAAGCTC | |
| TaTOC1-F | CAGCACTTGCAAAATTCAGGC | |
| TaTOC1-R | AACTCAACGTCCCTGGAGGAT | |
| TaPRR37-F | CCACTGCATGTACCAAGTTATC | |
| TaPRR37-R | ATGTCCTTGCAGACCTCGTGGT | |
| TaPRR73-F | TATCTCCAGGACATGCAGAGC | |
| TaPRR73-R | AGCACCATTTGACAGGCAACT | |
| TaPRR95-F | AGTGCGGCTACCACGTTTCTG | |
| TaPRR95-R | GCTAACTGCATCATTCGAAGAC | |
| LUX-F | TGCACAAGCGGTTCGTGGAGGT | |
| LUX-R | TGGAGAGGCCCTGCATCCGCTT | |
| TraesCS1D02G226100-F | TGCTCTTGGTGCGAGTTACA | |
| TraesCS1D02G226100-R | TACCGGTGCTCAGGAGGTAG | |
| TraesCS3B02G186700-F | GTCTGGAAGCATGGGAGAAT | |
| TraesCS3B02G186700-R | GGCTTTCCGTGAAATACTCG | |
| TraesCS1D02G295200-F | ATGACACAATCAAACCCG | |
| TraesCS1D02G295200-R | TCAATTGAAGAAGTAATTG | |
| TraesCS2B02G491700-F | CTCGTTCGGTGCATGGTA | |
| TraesCS2B02G491700-R | CTTGTCTGCTCTGCTGTG | |
| Actin-F | GTTGGTGATGAGGCCCAATC | |
| Actin-R | GTGCTACACGGAGCTCATTG | |
| **ChIP-PCR** | | |
| Primers used in Figure.2 | | |
| TaCCA1-7A-H3K4me3-F | GTGTGTGCCTGGTCGCTCT (-5,142 bp ⁓ -5,123 bp) | |
| TaCCA1-7A-H3K4me3-R | CCCCCCTCTCCAACAACAG (-5,022 bp ⁓ -5,003 bp) | |
| TaCCA1-7A-H3K9ac-F | GTGTGTGCCTGGTCGCTCT (-5,142 bp ⁓ -5,123 bp) | |
| TaCCA1-7A-H3K9ac-R | CCCCCCTCTCCAACAACAG (-5,022 bp ⁓ -5,003 bp) | |
| TaCCA1-7A-H3K36me3-F | GTGTGTGCCTGGTCGCTCT (-5,142 bp ⁓ -5,123 bp) | |
| TaCCA1-7A-H3K36me3-R | CCCCCCTCTCCAACAACAG (-5,022 bp ⁓ -5,003 bp) | |
| TaCCA1-7B-H3K4me3-F | TGTTGGAGAGGGGGGATTAC (-4,942 bp ⁓ -4,923 bp) | |
| TaCCA1-7B-H3K4me3-R | AAAAGGGCACAGAAGACGG (-4,820 bp ⁓ -4802 bp) | |
| TaCCA1-7B-H3K9ac-F | TGTTGGAGAGGGGGGATTAC (-4,942 bp ⁓ -4,923 bp) | |
| TaCCA1-7B-H3K9ac-R | AAAAGGGCACAGAAGACGG (-4,820 bp ⁓ -4802 bp) | |
| TaCCA1-7B-H3K36me3-F | TGTTGGAGAGGGGGGATTAC (-4,942 bp ⁓ -4,923 bp) | |
| TaCCA1-7B-H3K36me3-R | AAAAGGGCACAGAAGACGG (-4,820 bp ⁓ -4802 bp) | |
| TaCCA1-7D-H3K4me3-F | TAAACAACTTCAGAGCACCAAC ( -4,578 bp ⁓ -4,557 bp) | |
| TaCCA1-7D-H3K4me3-R | CAACTGCTTGCAGCTAGAAA (-4,707bp ⁓ -4,688 bp) | |
| TaCCA1-7D-H3K9ac-F | TAAACAACTTCAGAGCACCAAC ( -4,578 bp ⁓ -4,557 bp) | |
| TaCCA1-7D-H3K9ac-R | CAACTGCTTGCAGCTAGAAA (-4,707bp ⁓ -4,688 bp) | |
| TaCCA1-7D-H3K36me3-F | ATCCCTGCCACCCACCTCA (-5,658 bp ⁓ -5,640 bp) | |
| TaCCA1-7D-H3K36me3-R | CACGCTTGTTCCTCCCGCT (-5,539 bp ⁓ -5,520 bp) | |
| Primers used in Figure.S4 | | |
| TaCCA1-7A-H3K4me3-2F | GGAGAGGGGGGATTACGGAT (-5,015 bp ⁓ -4,996 bp) | |
| TaCCA1-7A-H3K4me3-2R | GAAAAGGGCACAGAAGACGG (-4,896 bp ⁓ -4,877 bp) | |
| TaCCA1-7A-H3K9ac-2F | GGAGAGGGGGGATTACGGAT (-5,015 bp ⁓ -4,996 bp) | |
| TaCCA1-7A-H3K9ac-2R | GAAAAGGGCACAGAAGACGG (-4,896 bp ⁓ -4,877 bp) | |
| TaCCA1-7A-H3K36me3-2F | CGCCAAACATACTCCTTCAATC (-3,789 bp ⁓ -3,768 bp) | |
| TaCCA1-7A-H3K36me3-2R | CGCTTGCCTGCTCTTCTATGA (-3,641 bp ⁓ -3,621 bp) | |
| TaCCA1-7B-H3K4me3-2F | TGCCTGGTCGGTCTCTGTAGT (-4,942 bp⁓-4,923 bp) | |
| TaCCA1-7B-H3K4me3-2R | ATTCGTAATCCCCCCTCTCC (-4,820bp ⁓ -4802 bp) | |
| TaCCA1-7B-H3K9ac-2F | TGCCTGGTCGGTCTCTGTAGT (-4,942 bp⁓-4,923 bp) | |
| TaCCA1-7B-H3K9ac-2R | ATTCGTAATCCCCCCTCTCC (-4,820bp ⁓ -4802 bp) | |
| TaCCA1-7B-H3K36me3-2F | TGCCTGGTCGGTCTCTGTAGT (-4,942 bp⁓-4,923 bp) | |
| TaCCA1-7B-H3K36me3-2R | ATTCGTAATCCCCCCTCTCC (-4,820bp ⁓ -4802 bp) | |
| TaCCA1-7D-H3K4me3-2F | CTTCTGTGCCCTTTTCTTGC (-4,961 bp ⁓ -4942 bp) | |
| TaCCA1-7D-H3K4me3-2R | CGGGTAGAAGAAGCCAAACT (-4,853 ⁓ -4,834 bp) | |
| TaCCA1-7D-H3K9ac-2F | CTTCTGTGCCCTTTTCTTGC (-4,961 bp ⁓ -4,942 bp) | |
| TaCCA1-7D-H3K9ac-2R | CGGGTAGAAGAAGCCAAACT (-4,853 ⁓ -4,834 bp) | |
| TaCCA1-7D-H3K36me3-2F | CTTCTGTGCCCTTTTCTTGC (-4,961 bp ⁓ -4,942 bp) | |
| TaCCA1-7D-H3K36me3-2R | CGGGTAGAAGAAGCCAAACT (-4,853 ⁓ -4,834 bp) | |

The number in the bracket indicates primer position in the genomic region relative to the start codon

**Table S2. List of oligonucleotides used in BLI and EMSA.**

| **Gene name** |  | **Sequence (5′to 3′)** |
| --- | --- | --- |
| TraesCS1D02G226100 | DAP1-F | TTATATGCCTATATGATATTATGATTTATTTGGTTT |
|  | DAP1-R | AAACCAAATAAATCATAATATCATATAGGCATATAA |
|  | DAP1-M_F | TTATATGCCTATATacccgTATGATTTATTTGGTTT |
|  | DAP1-M_R | AAACCAAATAAATCATAcgggtATATAGGCATATAA |
| TraesCS1D02G295200 | DAP2-F | AAATAGAAAATTACTGATATTTCATCCTTGTGTGAT |
|  | DAP2-R | ATCACACAAGGATGAAATATCAGTAATTTTCTATTT |
|  | DAP2-M_F | AAATAGAAAATTACTacgcgTTCATCCTTGTGTGAT |
|  | DAP2-M_R | ATCACACAAGGATGAAcgcgtAGTAATTTTCTATTT |
| TraesCS2B02G491700 | DAP3-F | CTTCAGATTTGTTGATATTTTCATATTGCAGACAT |
|  | DAP3-R | ATGTCTGCAATATGAAAATATCAACAAATCTGAAG |
|  | DAP3-M_F | CTTCAGATTTGTTtgcggTTTCATATTGCAGACAT |
|  | DAP3-M_R | ATGTCTGCAATATGAAAccgcaAACAAATCTGAAG |
| TraesCS3B02G186700 | DAP4-F | AATAGAAAATTACTGATATTTCATCCTTATGAGATT |
|  | DAP4-R | AATCTCATAAGGATGAAATATCAGTAATTTTCTATT |
|  | DAP4-M_F | AATAGAAAATTACTtcccgTTCATCCTTATGAGATT |
|  | DAP4-M_R | AATCTCATAAGGATGAAcgggaAGTAATTTTCTATT |

EE elements are underlined. Mutated sequences are lower cases. F: forward strand; R: reverse strand; M-F/R: DNA in which EE site was mutated.

**Table S3.** **Binding affinity of MBP-TaCCA1-7D fusion protein to TaCCA1-7A and TaCCA1-7B.**

| **Sensor** | **protein** | **protein** | **KD (M)** | **kon(1/Ms)** | **kdis(1/s)** |
| --- | --- | --- | --- | --- | --- |
| SA | MBP-TaCCA1-7D | TaCCA1-7A | 2.78E-08 | 4.30E+04 | 1.20E-03 |
| SA | MBP-TaCCA1-7D | TaCCA1-7B | 3.6E-09 | 3.68E+05 | 1.33E-03 |

**Table S4.** **Mapping statistics of DAP-seq.**

|  | **CCA1-rep1** | **%** | | **CCA1-rep2** | **%** |
| --- | --- | --- | --- | --- | --- |
| Total reads | 8028430 |  | 38967883 | |  |
| Trimmed | 7965304 | 99.21 | 38728275 | | 99.39 |
| Aligned | 7614966 | 95.6 | 38628862 | | 99.74 |
| MAPQ>10 | 4190570 | 55.03 | 26743383 | | 69.23 |
| Remove dups | 3703905 | 88.39 | 22199341 | | 83.01 |

**Table S5.** **Distribution of DAP-seq peaks.**

| **Region** | **AATATC** | **non_AATATC** |
| --- | --- | --- |
| Genebody | 12545 | 3453 |
| Intergenic | 307931 | 106038 |
| TES | 11881 | 4249 |
| promoter-TSS | 15657 | 5589 |
| Total | 348014 | 119329 |

**Table S6.** **Binding affinity of MBP-TaCCA1-7D fusion protein to dsDNA DAP-1--DAP-4.**

| **Sensor** | **Protein** | **dsDNA** | **Ka(1/Ms)** | **Kd(1/s)** | **KD(M)** |
| --- | --- | --- | --- | --- | --- |
| SA | MBP-TaCCA1-7D | DAP-1 | 1.35E+05 | 2.42E-04 | 1.79E-09 |
| SA | MBP-TaCCA1-7D | DAP-2 | 1.37E+05 | 2.35E-04 | 1.72E-09 |
| SA | MBP-TaCCA1-7D | DAP-3 | 1.31E+05 | 2.56E-04 | 1.95E-09 |
| SA | MBP-TaCCA1-7D | DAP-4 | 9.13E+04 | 1.50E-04 | 1.64E-09 |
